# Rapid, Sensitive, Full-Genome Sequencing of Severe Acute Respiratory Syndrome Coronavirus 2

**DOI:** 10.3201/eid2610.201800

**Published:** 2020-10

**Authors:** Clinton R. Paden, Ying Tao, Krista Queen, Jing Zhang, Yan Li, Anna Uehara, Suxiang Tong

**Affiliations:** Centers for Disease Control and Prevention, Atlanta, Georgia, USA (C. Paden, Y. Tao, K. Queen, Y. Li, S. Tong); IHRC, Atlanta (J. Zhang);; Oak Ridge Institute for Science and Education, Oak Ridge, Tennessee, USA (A. Uehara)

**Keywords:** coronavirus disease, COVID-19, severe acute respiratory syndrome coronavirus 2, SARS-CoV-2, coronavirus, viruses, genomics, high-throughput nucleotide sequencing, whole-genome sequencing, respiratory infections, zoonoses

## Abstract

We describe validated protocols for generating high-quality, full-length severe acute respiratory syndrome coronavirus 2 genomes from primary samples. One protocol uses multiplex reverse transcription PCR, followed by MinION or MiSeq sequencing; the other uses singleplex, nested reverse transcription PCR and Sanger sequencing. These protocols enable sensitive virus sequencing in different laboratory environments.

In December 2019, severe acute respiratory syndrome coronavirus 2 (SARS-CoV-2), the etiologic agent of coronavirus disease 2019 (COVID-19), emerged in Wuhan, China. Since then, it has rapidly spread worldwide (*1*–*3*), causing 7,039,918 confirmed cases, including 404,396 deaths, in 188 countries or regions as of June 9, 2020 (*4*). Because SARS-CoV-2 has shown the capacity to spread rapidly and lead to a range of manifestations in infected persons, from asymptomatic infection to mild, severe, or fatal disease, it is essential to identify genetic variants to track spread and understand any changes in transmissibility, tropism, and pathogenesis.

We describe the design and use of 2 PCR-based methods for sequencing SARS-CoV-2 clinical specimens. The first is a multiplex PCR panel, followed by sequencing on either the Oxford Nanopore MinION apparatus (https://nanoporetech.com) or an Illumina MiSeq apparatus (https://www.illumina.com). When coupled with MinION sequencing, our protocol can be implemented outside a traditional laboratory and can be completed in a single workday, similar to previous mobile genomic surveillance of Ebola and Zika virus outbreaks (*5*,*6*). In addition, we provide a complementary singleplex, nested PCR strategy, which improves sensitivity for samples with lower viral load and is compatible with Sanger sequencing.

## The Study

On January 10, 2020, the first SARS-CoV-2 genome sequence was released online (*7*). That day, we designed 2 complementary panels of primers to amplify the virus genome for sequencing.

For the first panel, we used the PRIMAL primer design tool (*5*) to design multiplex PCRs to amplify the genome by using only a few PCRs (Appendix). The final design consists of 6 pools of primers optimized for sensitivity and assay flexibility. The amplicons average 550 bp with 100-bp overlaps to enable sequencing on either the Oxford MinION or Illumina MiSeq.

For the second panel, we designed sets of primers to generate nested, tiling amplicons across the SARS-CoV-2 genome (Appendix) for enhanced sensitivity in samples with lower viral loads. Each amplicon is 322–1,030 bp with an average overlap of 80 bp. These amplicons are designed to be amplified and sequenced individually on Sanger instruments but can also be pooled for sequencing on next-generation sequencing platforms.

To determine the sensitivity of each sequencing strategy, we generated a set of 6 ten-fold serial dilutions of a SARS-CoV-2 isolate (*8*). Virus RNA was diluted into a constant background of A549 human cell line total nucleic acid (RNaseP cycle threshold [C_t_] 29). We quantitated each dilution by using the Centers for Disease Control and Prevention SARS-CoV-2 real-time reverse transcription PCR for the nucleocapsid 2 gene (*9*). The 6 dilutions spanned C_t_ values from 22 to 37, corresponding to ≈2 × 10^0^ to 1.8 × 10^5^ copies. We amplified triplicate samples at each dilution by using the multiplex PCR pools. Next, we pooled, barcoded, and made libraries from amplicons of each sample by using the ligation-based kit and PCR barcode expansion kit (Appendix). MinION sequencing was performed on an R9.4.1 or R10.3 flow cell (Oxford) until we obtained >1–2 million raw reads. From those reads, 50%–60% of them could be demultiplexed. In addition, we sequenced these amplicons by using the Illumina MiSeq for comparison (Appendix).

**Figure 1:**
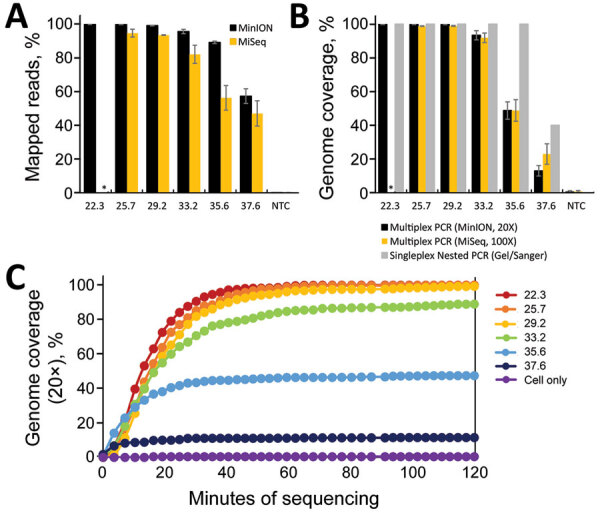
Limits of detection for sequencing severe acute respiratory syndrome coronavirus 2. Triplicate serial dilutions of virus isolate A12 (*8*) were amplified by using the singleplex or multiplex primer set. Multiplex amplicons were barcoded, library-prepped, and sequenced on an Oxford MinION apparatus (https://nanoporetech.com) or an Illumina MiSeq apparatus (https://www.illumina.com). A) Percentage of reads that map to the virus genome for each sample. B) Percentage of virus genome that is covered at >20× depth by the multiplex amplicons on the MinION (black) or >100× depth on the MiSeq (orange), or covered by the nested, singleplex amplicons (gray) (measured by presence or absence on a gel). C) Real-time analysis of MinION sequencing data. Each data point represents the average 20× genome coverage of three replicates. NTC, nontemplate controls (human cell nucleic acid carried through the PCR and library preparation). Asterisk (*) indicates that samples were not analyzed at that dilution.

For MinION sequencing, the reads were basecalled and analyzed by using an in-house read mapping pipeline (Appendix). For samples with C_t_ <29, we obtained >99% SARS-CoV-2 reads and >99% genome coverage at 20× depth, decreasing to an average of 93% genome coverage at C_t_ 33.2 and 48% at C_t_ 35 (Figure 1, panels A, B). Furthermore, we were able to obtain full genomes at >20× reading depth within the first 40–60 min of sequencing (Figure 1, panel C).

Consensus accuracy, including single-nucleotide polymorphisms and indels, is critical for determining coronavirus lineage and transmission networks. For high-consensus–level accuracy, we filtered reads based on length, mapped them to the reference sequence (GenBank accession no. RefSeq NC_045512), trimmed primers based on position, and called variants with Medaka ((https://github.com/nanoporetech/medaka) (Appendix). Each Medaka variant was filtered by coverage depth (>20×) and by the Medaka model-derived variant quality (>30). We used the variant quality score as a heuristic to filter remaining noise from the Medaka variants compared with Sanger-derived sequences. After these steps, the data approaches 100% consensus accuracy (Table 1). Identical results were found by using the R9.4.1 pore through samples with C_t_ values through 33.2. The larger deletions in some of the samples with C_t_ values >33.2 (Table 1) do not appear to be sequencing errors because they are also detected as minor populations within higher-titer samples.

**Table 1:** Genome consensus accuracy for sequencing severe acute respiratory syndrome coronavirus 2*

Virus titer (cycle threshold)	% Coverage, 20×†	Indels	Indel bases	Single-nucleotide polymorphisms	% Identity†
22.3	99.659	0	0	0	100
	99.722	0	0	0	100
	99.635	0	0	0	100
25.7	99.635	0	0	0	100
	99.615	0	0	0	100
	99.642	0	0	0	100
29.2	99.508	0	0	0	100
	99.635	0	0	0	100
	99.615	0	0	0	100
33.2	93.024	1	1	0	100
	93.603	2	35	0	100
	87.894	0	0	0	100
35.6	41.653	1	1	0	100
	51.266	0	0	1	99.993
	50.821	1	15	2	99.987
37.6	14.634	0	0	1	99.977
	9.317	0	0	0	100
	12.363	0	0	0	100

*Because the 5′ and 3′ ends are primer sequences, 100% coverage is not possible. †Percentage of covered bases identical to reference sequence, excludes indels and low-coverage bases.

In the MiSeq data, we observed a similar trend in percent genome coverage at 100× depth, and a slightly lower percentage mapped reads compared with Nanopore data (Figure 1, panels A, B). Increased read depth using the MiSeq potentially enables increased sample throughput. However, the number of available unique dual indices limits actual throughput.

For the nested, singleplex PCR panel, we amplified the same serial dilutions with each nested primer set (Appendix). The endpoint dilution for full-genome coverage is a C_t_ ≈35 (Figure 1, panel B). At the C_t_ 37 dilution, we observed major amplicon dropout; at this dilution, there are <10 copies of the genome on average/reaction.

These protocols enabled rapid sequencing of initial clinical cases of infection with SARS-CoV-2 in the United States. For these cases, we amplified the virus genome by using PCR and sequencing the amplicons by using the MinION and Sanger instruments to validate MinION consensus accuracy. The MinION produced full-length genomes in <20 min of sequencing, and Sanger data was available the following day.

**Figure 2:**
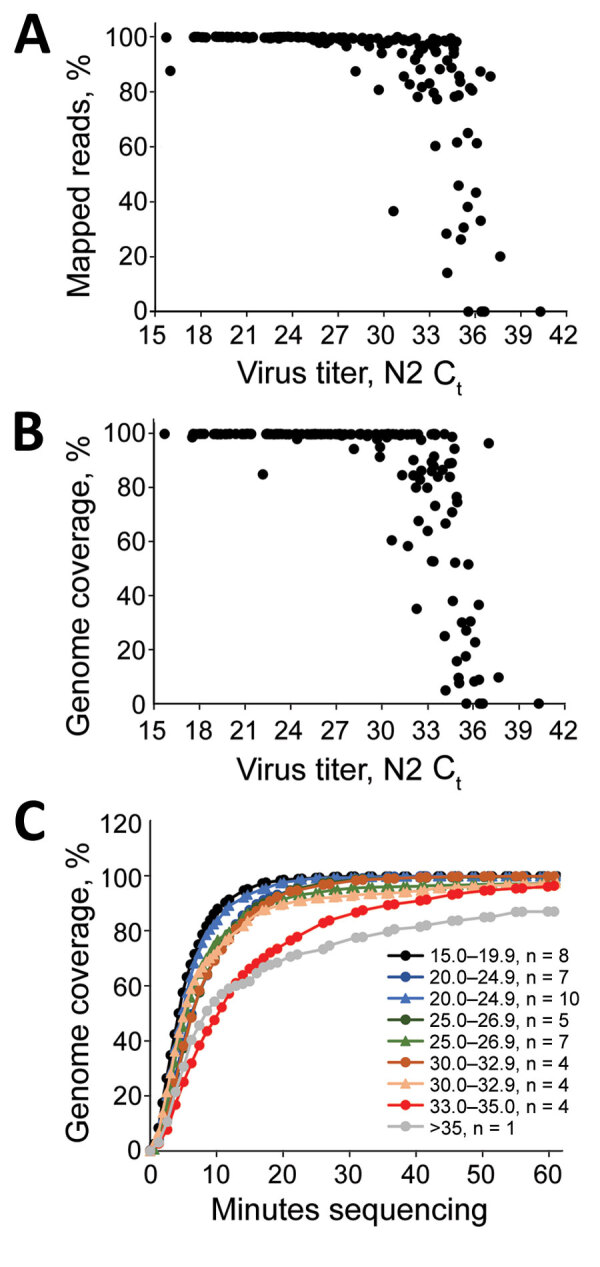
Sequencing of severe acute respiratory syndrome coronavirus 2 clinical samples. A, B) Percentage mapped (A) and percentage genome coverage (B) for 167 clinical severe acute respiratory syndrome coronavirus 2 samples amplified by using a multiplex PCR strategy and sequenced on the MinION apparatus (https://nanoporetech.com). C) Time-lapse of 20× genome coverage obtained for clinical specimens at the indicated cycle threshold values. Data points indicate average coverage over time for various samples and grouped by run and the indicated C_t_ values. C_t_, cycle threshold; N2, nucleoprotein 2.

We used the multiplex PCR strategy for subsequent SARS-CoV-2 clinical cases (n = 167) with C_t_ values ranging from 15.7 to 40 (mean 28.8, median 29.1). In cases with a C_t_ <30, we observed an average of 99.02% specific reads and 99.2% genome coverage at >20× depth (Figure 2, panels A, B). Between C_t_ 30 and 33, genome coverage varied by sample, and decreased dramatically at higher C_t_ values, analogous to the isolate validation data. For these samples, we multiplexed 20–40 barcoded samples/flowcell. Enough data are obtained with 60 min of MinION sequencing for most samples, although for higher titer samples, 10–20 min of sequencing is sufficient (Figure 2, panel C).

Up-to-date primer sequences, protocols, and analysis scripts are available on GitHub (https://github.com/CDCgov/SARS-CoV-2_Sequencing/tree/master/protocols/CDC-Comprehensive). Data from this study is deposited in the National Center for Biotechnology Information Sequence Read Archive (BioProjects PRJNA622817 and PRJNA610248).

## Conclusions

Full-genome sequencing is a critical tool in understanding emerging viruses. Initial sequencing of SARS-CoV-2 showed limited genetic variation (*10**,**11*). However, some signature variants have been useful for describing the introduction and transmission dynamics of the virus (*12**,**13*; T. Bedford et al., unpub. data, https://doi.org/10.1101/2020.04.02.20051417; M. Worobey et al., unpub. data, https://doi.org/10.1101/2020.05.21.109322).

We provide 2 validated PCR target-enrichment strategies that can be used with MinION, MiSeq, and Sanger platforms for sequencing SARS-CoV-2 clinical specimens. These strategies ensure that most laboratories have access to >1 strategies.

The multiplex PCR strategy is effective at generating full genome sequences up to C_t_ 33. The singleplex, nested PCR is effective up to C_t_ 35, varying based on sample quality. The turnaround time for the multiplex PCR MinION protocol is ≈8 hours from nucleic acid to consensus sequence and that for Sanger sequencing is ≈14–18 hours (Table 2). The multiplex PCR protocols offer an efficient, cost-effective, scalable system, and add little time and complexity as sample numbers increase (Table 2). Results from this study suggest multiplex PCR might be used effectively for routine sequencing, complemented by singleplex, nested PCR for low-titer virus samples and confirmation sequencing.

**Table 2:** Comparison of input, time, and cost requirements for sequencing 1 or 96 specimens of severe acute respiratory syndrome coronavirus 2

Method	Input, μL*	1 sample			96 samples	
		Turnaround time	Approximate cost/sample†		Turnaround time	Approximate cost/sample†
Multiplex/MinION	10	6–8 h	$528.70		8–10 h	$35.88
Multiplex/MiSeq	10	30–68 h‡	$1,443.29		30–68 h‡	$57.87
Singleplex/Sanger	190	16–18 h	$354.40		17–19 d	$354.40

^*^Assumes a process with 200 μL of resuspended respiratory specimen (from a total of 2 mL), extracted, and eluted into 100 μL See **Appendix** for details. †Includes specific enzyme and reagent costs; excludes common laboratory supplies and labor costs. ‡Varies according to the sequencing kit used.

Appendix Additional information on rapid, sensitive, full-genome sequencing of severe acute respiratory syndrome coronavirus 2.

## References

[1] Holshue ML, DeBolt C, Lindquist S, Lofy KH, Wiesman J, Bruce H, et al.; Washington State 2019-nCoV Case Investigation Team. Washington State 2019-nCoV Case Investigation Team. First case of 2019 novel coronavirus in the United States. N Engl J Med. 2020;382:929–36. 10.1056/NEJMoa2001191 32004427 PMC7092802

[2] Patel A, Jernigan DB, Abdirizak F, Abedi G, Aggarwal S, Albina D, et al.; 2019-nCoV CDC Response Team. 2019-nCoV CDC Response Team. Initial public health response and interim clinical guidance for the 2019 novel coronavirus outbreak—United States, December 31, 2019–February 4, 2020. MMWR Morb Mortal Wkly Rep. 2020;69:140–6. 10.15585/mmwr.mm6905e1 32027631 PMC7004396

[3] Wang C, Horby PW, Hayden FG, Gao GF. A novel coronavirus outbreak of global health concern. Lancet. 2020;395:470–3. 10.1016/S0140-6736(20)30185-9 31986257 PMC7135038

[4] World Health Organization. Coronavirus disease 2019 (COVID-19) situation report 141 [cited 2020 Jun 9]. https://www.who.int/emergencies/diseases/novel-coronavirus-2019/situation-reports

[5] Quick J, Grubaugh ND, Pullan ST, Claro IM, Smith AD, Gangavarapu K, et al. Multiplex PCR method for MinION and Illumina sequencing of Zika and other virus genomes directly from clinical samples. Nat Protoc. 2017;12:1261–76. 10.1038/nprot.2017.066 28538739 PMC5902022

[6] Quick J, Loman NJ, Duraffour S, Simpson JT, Severi E, Cowley L, et al. Real-time, portable genome sequencing for Ebola surveillance. Nature. 2016;530:228–32. 10.1038/nature16996 26840485 PMC4817224

[7] Holmes EC, Novel YZ. 2019 coronavirus genome, 2020 [cited 2020 Apr 5]. http://virological.org/t/novel-2019-coronavirus-genome/319

[8] Harcourt J, Tamin A, Lu X, Kamili S, Sakthivel SK, Murray J, et al. Severe Acute Respiratory Syndrome Coronavirus 2 from Patient with Coronavirus Disease, United States. Emerg Infect Dis. 2020;26:1266–73. 10.3201/eid2606.200516 32160149 PMC7258473

[9] COVID-19 Investigation Team. Clinical and virologic characteristics of the first 12 patients with coronavirus disease 2019 (COVID-19) in the United States. Nat Med. 2020;26:861–8. 10.1038/s41591-020-0877-5 32327757 PMC12755114

[10] Andersen K. Clock and TMRCA based on 27 genomes, 2020 [cited 2020 Jan 25]. http://virological.org/t/clock-and-tmrca-based-on-27-genomes/347

[11] Lu R, Zhao X, Li J, Niu P, Yang B, Wu H, et al. Genomic characterisation and epidemiology of 2019 novel coronavirus: implications for virus origins and receptor binding. Lancet. 2020;395:565–74. 10.1016/S0140-6736(20)30251-8 32007145 PMC7159086

[12] Andersen KG, Rambaut A, Lipkin WI, Holmes EC, Garry RF. The proximal origin of SARS-CoV-2. Nat Med. 2020;26:450–2. 10.1038/s41591-020-0820-9 32284615 PMC7095063

[13] Deng X, Gu W, Federman S, du Plessis L, Pybus OG, Faria N, et al. Genomic surveillance reveals multiple introductions of SARS-CoV2 into northern California. Science. 2020 Jun 8:eabb9263. 10.1126/science.abb9263 PMC7286545 32513865

