# Supplementary material for: Rapid, Sensitive, Full-Genome Sequencing of Severe Acute Respiratory Syndrome Coronavirus 2
Source: Emerg Infect Dis. 2020 Oct;26(10):2401–5. doi: 10.3201/eid2610.201800 (PMC7510745; doi:10.3201/eid2610.201800)
Supplement: Appendix — Additional information on rapid, sensitive, full-genome sequencing of severe acute respiratory syndrome coronavirus 2. [file 20-1800-Techapp-s1.pdf]

Article DOI: https://doi.org/10.3201/eid2610.20.1800

Rapid, Sensitive, Full-Genome Sequencing of Severe Acute Respiratory Syndrome Coronavirus 2

Appendix

The following pages describe 2 validated protocols for generating high-quality, full-length severe acute respiratory syndrome coronavirus 2 genomes from primary samples. One protocol uses multiplex reverse transcription PCR, followed by MinION or MiSeq sequencing; the other uses singleplex, nested reverse transcription PCR and Sanger sequencing.

Protocols for SARS-CoV-2 sequencing

Pathogen Discovery Team
NCIRD/DVD/RVB
Centers for Diseases Control and Prevention

Table of Contents

Disclaimers 4

Application Notes 5

Singleplex nested RT-PCR 6

Protocol Notes 6

Required Reagents 6

Procedure 6

1. First round of RT-PCR 6
2. Second round of semi-nested or nested PCR 7

Sanger sequencing 8

Required Reagents 8

Procedure 8

Multiplex PCR 9

Protocol Notes 9

Required reagents 9

Procedure 9

1. Generate primer pools 9
2. First-strand synthesis 9
3. Multiplex PCR 10

Nanopore Sequencing 11

Protocol Notes 11

Procedure 11

1. Barcode amplicons 11
2. Prepare Nanopore Ligation-based Library 12
3. Load MinION and sequence 13
4. Generate consensus sequences from MinION data 14
5. Quality control and analysis suggestions 15

Illumina Library Preparation and Sequencing 16

Protocol Notes 16

Required Reagents 16

Procedure for Library Preparation 16

1. Fragmentation and End Repair 16
2. Adapter Ligation 17
3. PCR enrichment of Adapter-Ligated DNA 17

4. Sizing and quantitation 18
MiSeq sequencing 20
Protocol Notes 20
Required Reagents 20
Procedure 20
1. Dilute and Pool Libraries 20
2. Denature Libraries 20
3. Load and Run MiSeq 21
4. Generation of consensus sequences from MiSeq data 22
Appendix A – Singleplex PCR Primers 23
Appendix B – Sequencing Primers 25
Appendix C – Plate Setup for Nested PCR and Sanger Sequencing 27
Appendix D – Multiplex PCR Primers 29
Appendix E – AMPure XP bead clean-up 32
Appendix F – Quantitation using Qubit 33
Required reagents 33
Procedure 33
Appendix G – CENTRI-SEP 96 Protocol 34

Disclaimers

The findings and conclusions in this report have not been formally disseminated by the Centers for Disease Control and Prevention and should not be construed to represent any agency determination or policy.

The protocols described here are for research purposes only and should not be used in place of approved diagnostic testing.

Application Notes

Validation specimen submission extraction, and quantitation

1. For clinical specimens, CDC requested that submitting labs submit upper respiratory swabs in 2-3 mL viral transport medium (VTM), according to the guidelines detailed at: https://www.cdc.gov/coronavirus/2019-ncov/lab/guidelines-clinical-specimens.html.
2. Extraction of respiratory specimens in was performed using the QIAamp Viral RNA Mini Kit (QIAGEN). 200 uL of specimen VTM was used for each extraction and eluted from the column in 100 uL RNase-free water.
3. Samples for validation were quantitated with CDC N2 qRT-PCR assay, detailed at https://www.cdc.gov/coronavirus/2019-ncov/lab/rt-pcr-panel-primer-probes.html

General Guidelines

1. Multiplex PCR Protocol is effective for Ct < 33.
2. Singleplex PCR is effective for some samples up to Ct 35. These are also useful for fill in reactions.
3. For the full Singleplex/Sanger protocol, two nucleic acid extractions (400 uL raw sample) will be needed.

Singleplex nested RT-PCR

Protocol Notes

To complete this protocol, 190 uL of extracted template is needed. For samples between Ct 27 and 35, two rounds of nested RT-PCR are recommended; for samples up to Ct 27, one round of RT-PCR is recommended. The resulting PCR products can be individually proceeded with Sanger sequencing, or they can be pooled for Oxford Nanopore or Illumina sequencing, depending on the number of samples and availability of sequencing platforms.

See Appendix C for recommended plate setups.

Required Reagents

Company	Product	Catalog number
Thermo (Invitrogen)	Superscript III one-step RT-PCR with Platinum Taq High Fidelity DNA polymerase	12574035
Sigma Aldrich (Roche)	Protector RNase inhibitor	3335402001
Takara	LA Taq DNA polymerase with GC buffer	RR02AG
	Nuclease Free water	
	50 uM Primers	

Procedure

1. First round of RT-PCR

1.1. Prepare the first-round master mix as below. Please note, the protocol is generic as all 38 primer pairs require the same master mix (see Appendix A). For each SARS-CoV-2 sample to be sequenced, 38 individual PCR reactions are required.

Component	Volume (uL)
Water	1.75
2x Buffer (2.4mM $MgSO_4$)	12.5
5mM $MgSO_4$	4.5
50uM Primer For	0.25
50uM Primer Rev	0.25
RNase Inhib. 40U/uL	0.25
SSIII / Platinum Taq high fidelity	0.5
Pre-mix	20
Template (RNA)	5
Total	25

1.2. Add 5uL of RNA template to each of the 38 PCR reactions. Spin tubes/plates down and proceed to PCR.

1.3. Perform first round PCR with the cycling parameters as below.

60°C	1 minutes	Decrease 0.5 C°/sec
50°C	30 minutes	
94°C	15 seconds	40 cycles
55°C	15 seconds	
72°C	1 minutes	
72°C	7 minutes	
4°C	Hold	

2. Second round of semi-nested or nested PCR

2.1. After first round RT-PCR is complete, prepare the master mix for 2nd round of semi-nested or nested PCR as below. Please note, the protocol is generic as all 38 second round primer pairs require the same master mix. Primer information is located in Appendix A. For the 2nd round of semi-nested- or nested PCR, there are 38 individual PCR reactions for each sample to be sequenced.

Component	Volume (uL)
Water	5.75
2× GCBuffer I	12.5
dNTP Mixture (2.5 mM each)	4
50uM Primer For	0.25
50uM Primer Rev	0.25
TaKaRa LA Taq™ (5 units/µl)	0.25
Pre-mix	23
Template (1R product)	2
Total	25

2.2. Add 2 uL of the corresponding first round PCR product to the second round PCR master mix. Spin tubes/plates down and proceed to PCR.

2.3. Perform second round PCR with the cycling parameters as below.

94°C	3 minutes	
94°C	15 seconds	30 cycles
55°C	15 seconds	
72°C	1 minutes	
72°C	7 minutes	
4°C	Hold	

2.4. Following the completion of second round PCR, run 3 uL of all 38 PCR reactions on 1% agarose gels or fragment analyzer to check for amplification.

Sanger sequencing

Required Reagents

Company	Product	Catalog number
Thermo (Applied Biosystems)	ExoSap-It	78201.1.ML
Thermo (Applied Biosystems)	BigDye v3.1 cycle sequencing kit	4337455
Princeton Separations	Centri-sep 96 well plates	CS-963
	Nuclease Free water	
	5 uM Primers	

Procedure

1. Transfer 10 uL of each PCR reaction to new tubes/plate for ExoSap cleanup. Add 4 uL ExoSap-It to each PCR reaction (10 uL) and incubate at 37°C for 15 minutes, followed by 80°C for 15 minutes on a thermocycler.
2. Prepare sequencing master mix as below.
   Sequencing primers for each amplicon are listed in Appendix B

Component	Volume (uL)
Water	5.5
2x Buffer	2
5uM Primer	1
BigDye 3.1 enzyme	1
Pre-mix	9.5
Template (2R PCR product)	0.5
Total	10

3. Add 0.5 uL of corresponding ExoSap cleaned PCR product to each sequencing reaction mix. Spin tubes/plates down and proceed to sequencing PCR.
4. Perform sequencing PCR with the parameters listed below:

96°C	2 minutes	
96°C	30 seconds	30 cycles
50°C	15 seconds	
60°C	3 minutes	
4°C	Hold	

5. Following sequencing PCR, clean-up of sequencing reactions is performed with Centri-Sep 96-well plates following the manufacturer's instructions (Appendix G) with one addition. 20 uL nuclease free water is added to the 96-well collection plate prior to the final spin.
6. The 96-well collection plate with the cleaned sequencing sample plus water is loaded onto the ABI sequencer.
7. Sequencher 5.4 is used for data analysis of Sanger PCR data.

Multiplex PCR

Protocol Notes

This protocol uses 10 uL of template for each sample. The pooled, multiplexed PCR products can be followed with nanopore sequencing or Illumina MiSeq sequencing depending on the number of samples and available sequencing platforms. We have been able to sequence full genomes reliably under Ct 30, and depending on the sample, up to Ct 33.

This protocol was adapted from Quick J et al. *Nat Protoc*. 2017 Jun;12(6):1261-1276.

Required reagents

Company	Product	Catalog number
Thermo Fisher (Invitrogen)	SuperScript IV 1st strand synthesis system	18091200
NEB	NEBNext Q5 Hot Start HiFi PCR Master Mix	M0543L
	Nuclease Free water	
	Primers	

Procedure

1. Generate primer pools

1.1. Prepare primers as 50 uM primer stocks.

1.2. Add an equal volume of each 50 uM primer stock to six 1.5mL Eppendorf tubes labeled as pool 1, 2, 3, 4, 5, and 6. Primers for each pool are listed in Appendix D.

1.3. Prepare 10 uM working concentration by diluting each pool 1:5 with nuclease free water.

2. First-strand synthesis

2.1. Mix the following components.

Component	Volume(uL)
RNA (template)	10
Random primer 25uM	2
dNTPs	1
Total	13

2.2. Denature the template-primer-dNTP mix at 65°C for 5 minutes.

2.3. Place on ice for 5 minutes.

2.4. Add the following components to the template-primer-dNTP mix:

Component	Volume (uL)
5x SSIV buffer	4
0.1 M DTT	1
RNAse inhibitor	1
SSIV RT (200 units/uL)	1
Total	20

2.5. Incubate in a thermal cycler at the following temperatures:
25°C 10 minutes, 50°C for 10 minutes, 85°C for 10 minutes, hold at 4°C.
2.6. Spin down. Can be stored at -20°C
2.7. Add 1 uL RNase H and incubate at 37°C for 20 minutes

3. Multiplex PCR

3.1. Mix the following components in 6 wells of a PCR plate or strip tube.

Component	Volume (uL)
NEBNext Q5 Hot Start HiFi PCR Master Mix	15
PCR grade water	10.2
Primer pool 1, 2, 3, 4, 5, or 6 (10uM)	1.8
Total	27

3.2. Add 3 uL of cDNA from above to each tube.
3.3. Run the following PCR program:

98°C	30 seconds	
98°C	15 seconds	40 cycles
65°C	5 minutes	
4°C	Hold	

Note: fewer cycles may be used, but 40 cycles is used to maximize detection of lower-titer samples.
3.4. Optional: Run a 2% agarose gel for each multiplexed PCR reaction pool 1, 2, 3, 4, 5, and 6 to check for specific bands of the correct size (0.4-0.6 kb).
3.5. Pool 20 uL from each of 6 tubes of multiplexed PCR reactions in a 0.3 mL tube in a PCR strip or a well in PCR plate (the total volume is 120 uL).
3.6. Add 1X ratio (120 uL) of AMPure XP beads to the PCR product pools.
3.7. Purify according to standard AMPure protocol (see Appendix E).
3.8. Elute in 80 uL water.
3.9. Quantitate 1 uL of cleaned PCR products using Qubit dsDNA HS kit (Appendix F).
3.10. Optional: Run a 2% agarose gel and load 3 uL of cleaned PCR products to check for specific bands of the correct size (0.4-0.6 kb).

Nanopore Sequencing

Protocol Notes

This protocol takes advantage of the multiplexing density afforded by the "PCR Barcoding Expansion 1-96" kit. This protocol is derived from Oxford Nanopore's protocols available at http://community.nanoporetech.com.

Required reagents for Nanopore barcoding and sequencing:

Company	Product	Catalog number
NEB	NEBNext Ultra II End-repair/dA tailing module	E7546
NEB	Blunt/TA ligase master mix	M0367
NEB	NEBNext Quick Ligation Module	E6056
TaKaRa	TaKaRa LA Taq DNA Polymerase with GC Buffer	RR02AG
Beckman Coulter	Agencourt Ampure XP Beads	A63880/A63881
Oxford Nanopore Technologies	Nanopore Ligation Sequencing Kit (1D)	SQK-LSK109
Oxford Nanopore Technologies	PCR Barcoding Expansion 1-96	EXP-PBC096
Oxford Nanopore Technologies	SpotON Flow Cell (R9.4.1)	FLO-MIN106D
Oxford Nanopore Technologies	MinION	MinION Mk1B

Procedure

1. Barcode amplicons

1.1. Mix the following components:

Component	Volume (uL)
500 ng amplicon DNA	25
Ultra II end-prep reaction buffer	3.5
Ultra II end-prep enzyme mix	1.5
Total	30

1.2. Incubate at 20°C for 10 minutes, 65°C for 5 minutes, hold at 4°C.
1.3. Add 1X ratio (30 uL) AMPure XP beads.
1.4. Purify according to standard AMPure protocol (Appendex E).
1.5. Elute the DNA target from the beads with 17 uL water.
1.6. Optional: quantitate 1 uL of cleaned end-prep DNA using Qubit dsDNA HS kit (Appendex F)
1.7. Mix the following components:

Component	Volume (uL)
Cleaned end-prep DNA	15
Barcode Adapter	10
Blunt/TA ligase master mix	25
Total	50

1.8. Incubate at 20°C for 10 minutes.
1.9. Add 1X ratio (50 uL) AMPure XP beads.
1.10. Purify according to standard AMPure protocol (Appendix E).
1.11. Elute the DNA in 12 uL water.

1.12. Transfer eluate into new PCR plate or well
1.13. Quantitate 1 uL of ligated DNA according to the protocol (Appendix F).
1.14. Mix the following components:

Component	Volume(uL)
30ng adapter-ligated DNA	x
PCR Barcode primer (one of BC1-BC96)	1
2x GC Buffer I	25
dNTP mix (10mM)	8
TaKaRa LA Taq (5U/uL)	0.5
Water	50 – x
Total	50

1.15. Mix by pipetting and spin down
1.16. Run the following PCR program:

95°C	3 minutes	
95°C	15 seconds	18 cycles
62°C	15 seconds	
72°C	1 minutes	
72°C	7 minutes	
4°C	Hold	

1.17. Add 1X ratio (50 uL) of AMPure XP beads.
1.18. Purify according to standard AMPure protocol (Appendix E).
1.19. Elute the DNA target from the beads with 25 uL water.
1.20. Quantitate 1 uL cleaned, barcoded PCR products with Qubit dsDNA HS kit (Appendix F).

2. Prepare Nanopore Ligation-based Library

2.21. Pool the barcoded PCR products equally by mass.
2.22. Prepare LSK109 ligation-based libraries by mixing the following components:

Component	Volume(uL)
1 ug pooled barcoded sample	x
DNA CS	1
Ultra II End-prep reaction buffer	7
Ultra II End-prep enzyme mix	3
Water	49-x
Total	60

2.23. Incubate at 20°C for 10 minutes, 65°C for 5 minutes, hold at 4°C.
2.24. Add 1X ratio (60 uL) of AMPure XP beads.
2.25. Purify according to standard AMPure protocol (Appendix E).
2.26. Elute the DNA target from the beads with 62 uL water.
2.27. To ligate sequencing adapters, mixing the following components:

Component	Volume(uL)
End-repaired DNA from previous step	60
Ligation buffer (LNB)	25
NEBNext Quick T4 DNA Ligase	10
Adapter Mix (AMX)	5
Total	100

2.28. Incubate 10 minutes at 20°C
2.29. Add 0.8X ratio (80 uL) of AMPure XP beads
2.30. Purify according to standard AMPure protocol (Appendix E).
2.31. Elute the DNA target from the beads with 15 uL water
2.32. Quantitate 1 uL clean, prepared library with Qubit dsDNA HS kit (Appendix F).

3. Load MinION and sequence

3.33. Set up the MinION flow cell and host computer, including MinKNOW software.
3.34. Open the MinKNOW GUI from the desktop icon and establish a local connection.
3.35. Inset flow cell into MinION.
3.36. Click “Check Flow Cells” at the bottom of the screen then click “Start test.” Check the number of active pores available. When the check is complete, it is reported in the Notification panel. Check to ensure it has enough pores for a good sequencing run (warranty for flow cells: 800 nanopores or above checked within 5 days of receipt).
3.37. Thaw the Sequencing Buffer (SQB), Loading Beads (LB), Flush Tether (FLT) and one tube of Flush Buffer (FB) at room temperature before placing the tubes on ice.
3.38. Thoroughly mix the Sequencing Buffer (SQB) and Flush Buffer (FB) tubes by vortexing,
3.39. Spin down the Flush Tether (FLT) tube, mix by pipetting, and return to ice.
3.40. Open the lid of the nanopore sequencing device and slide the flow cell's priming port cover clockwise90 degrees. (The following steps are demonstrated at https://youtu.be/CC11Jlydqrc)
3.41. Set a P1000 pipette to 200 uL, insert the tip into the priming port, turn the wheel until the dial shows 220-230 uL, or until you can see a small volume of buffer entering the pipette tip. Do not remove more than this.
3.42. Visually check that there is continuous buffer from the priming port across the sensor array.
3.43. Prepare the flow cell priming mix: add 30 uL of thawed and mixed Flush Tether (FLT) directly to the tube of thawed and mixed Flush Buffer (FB), and mix by pipetting up and down.
3.44. Load 800 uL of the priming mix into the flow cell via the priming port, avoiding the introduction of air bubbles.
3.45. Wait for 5 minutes.
3.46. Thoroughly mix the contents of the Loading Beads (LB) by pipetting.
3.47. Prepare library for loading my mixing:

Component	Volume (uL)
Sequencing Buffer (SQB)	37.5
Loading Beads (LB), mixed immediately before use	25.5
150-200 ng DNA Library	12
Total	75

3.48. Gently lift the SpotON sample port cover to make the SpotON sample port accessible.
3.49. Load 200 µl of the priming mix into the flow cell via the priming port (not the SpotON sample port), avoiding the introduction of air bubbles.
3.50. Mix the prepared library gently by pipetting up and down just prior to loading.

3.51. Add 75 uL of sample to the flow cell via the SpotON sample port in a dropwise fashion. Ensure each drop flows into the port before adding the next.

3.52. Gently replace the SpotON sample port cover, making sure the bung enters the SpotON port, close the priming port and replace the MinION lid.

3.53. Start the sequencing run using the MinKNOW software.

4. Generate consensus sequences from MinION data

There are many considerations for generating high-quality consensus data from the MinION. Here are some suggestions for basecalling based on our experience.

Software:

Software	Source URL
Guppy 3.4.1+	https://community.nanoporetech.com/downloads
Medaka 0.11.5	https://github.com/nanoporetech/medaka
Minimap2 2.17 (r941)	https://github.com/lh3/minimap2
SAMtools 1.9	http://www.htslib.org/
BCFtools 1.9	http://www.htslib.org/
BAMClipper	https://github.com/tommyau/bamclipper
cutadapt 2.3+	https://github.com/marcelm/cutadapt
vcf_mask_lowcoverage.pl	https://github.com/CDCgov/SARS-CoV-2_Sequencing
IGV	http://software.broadinstitute.org/software/igv/

Example commands below have user-supplied variable names bold. You will need to customize the details to your environment.

4.1. Basecalling
Basecalling may also be done using MinKNOW software. If so, you may skip the Guppy basecalling step.

```
# Run Guppy
guppy_basecaller --input_path $rundir --save_path $outputdir -r \
    --config na_r9.4.1_450bps_hac.cfg --barcode_kits EXP-PBC096 \
    --trim_barcodes --require_barcodes_both_ends
# Combine all the output fastq files
mkdir $outputdir/fastq
find $outputdir -name "*.fastq" |while read infile; do
      if [[ $i =~ barcode|unclassified  ]]; then
         outfile=$(grep -Eo "barcode..|unclassified" <<< $infile).fastq
         outfile="fastq/$outfile"
         cat $infile >> $outfile
      fi
done
```

4.2. Filter on quality and length
Filtering out low quality sequence, as well as unexpectedly long and short reads helps tremendously on off-target mapping affecting consensus quality.

```
cutadapt -j $threads -m 300 -M 1200  -q 15 -o $fastqfiltered $fastqfile
```

4.3. Mapping

Download reference sequence from GenBank: MN908947.3

```
minimap2 -L -a -x map-ont -t 12 MN908947.fasta $ fastqfiltered > $samfile
samtools view -b $samfile | samtools sort - -o $bamfile
samtools index $bamfile
```

4.4. Clip primers

This step requires a BEDPE file describing the positions of the primers. It is available at https://github.com/CDCgov/SARS-CoV-2_Sequencing

BAMClipper by default will output at file with the suffix "primerclipped.bam."

Clipping by position allows only primers near the beginning of a read to be trimmed (rather than genuine sequence in the middle of a read), and it is faster than sequence-based trimming (e.g. Porechop).

```
cd $outputdir
bamclipper.sh -b $bamfile -p SC2_200324.bedpe -n 12 -u 80 -d 80
```

4.5. Generate VCF and consensus sequences

Medaka is very lenient with calling variants. We generally require a variant quality score of >= 30 and depth of coverage >= 20 to call a variant. Below 20X coverage, we call an 'N.'

The script to automate the filtering is available at https://github.com/CDCgov/SARS-CoV-2_Sequencing

```
# Generate Medaka VCF File
medaka consensus --model r941_min_high_g344 --threads 12 \ $primerclippedbamfile
$primerclippedbamfile.hdf
medaka variant MN908947.fasta $primerclippedbamfile.hdf $vcf
# Filter variants and generate consensus sequence
vcf_mask_lowcoverage.pl --bam $primerclippedbamfile  \
   --reference MN908947.fasta --vcf $vcf --consout $consensusfasta \
   --depth 20 --qual 30
```

5. Quality control and analysis suggestions

5.6. Watch out for 1-base insertions/deletions. Though consensus calling has improved considerably, there are residual errors. There are several stretches in SARS-CoV-2 that have homopolymers long enough to be problematic

5.7. Do not ignore other deletions. There have been several deletions reported (3, 9, 15, 33bp, 384bp, etc), so keep in mind the difference between a potential real indel and missing amplicon or nanopore error.

5.8. IGV can be useful for examining the "believability" of variants However, some of these 1-2bp indels appear in the reads, but they cannot be confirmed by Illumina or Sanger sequencing. These are either unlucky PCR bias or systematic sequencing error.

Illumina Library Preparation and Sequencing

Protocol Notes

Starting Material: 100 pg–250 ng DNA. We recommend that the DNA be in 1X TE (10 mM Tris pH 8.0, 1 mM EDTA), however, 10 mM Tris pH 7.5–8, low EDTA TE or water are also acceptable. If the input DNA is less than 26 µl, add TE (provided) to a final volume of 26 µl. This protocol is adapted from the NEBNext Ultra II FS protocol, which can be found in its entirety at http://www.neb.com.

For sizing, other devices, such as the 2100 BioAnalyzer, 5200 FragmentAnalyzer, QIAxcel, or LabChipGX may also be used. These vary in quantitation accuracy, so fluorometric quantitation with Qubit (or similar instrument) or qPCR is recommended.

Required Reagents

Company	Product	Catalog number
New England Biolabs (NEB)	NEBNext Ultra II FS DNA Library Prep Kit for Illumina	E7805S/E7805L
New England Biolabs (NEB)	NEBNext® Multiplex Oligos for Illumina (96 Unique Dual Index Primer Pairs)	E6440S/E6440L
Beckman Coulter	Agencourt Ampure XP Beads	A63880/A63881
	10mM Tris-HCl, pH 8.0	
	Molecular biology grade ethanol	
	Nuclease-free water	
Agilent	High Sensitivity D1000 screen tape	5067-5584
Agilent	High Sensitivity D1000 reagents	5067-5585

Procedure for Library Preparation

1. Fragmentation and End Repair

1.1. Ensure that the Ultra II FS Reaction Buffer is completely thawed. If a precipitate is seen in the buffer, pipette up and down several times to break it up, and quickly vortex to mix. Place on ice until use.

1.2. Vortex the Ultra II FS Enzyme Mix 5-8 seconds prior to use and place on ice.

1.3. Add the following components to a 0.2 ml thin wall PCR tube on ice

Component	Volume (uL)
NEBNext Ultra II FS Enzyme Mix (yellow tube)	2
NEBNext Ultra II FS Reaction Buffer (yellow tube)	7
DNA (pooled PCR amplicons)	26
Total	35

1.4. Vortex the reaction for 5 seconds and briefly spin down. Place in a thermocycler with the heated lid set to ≥75°C and run the following program:

37°C for 7 minutes, 65°C for 30 minutes, 4°C hold indefinitely

2. Adapter Ligation

2.1. Determine dilution for adapter if necessary, see table below. Dilute the NEBNext Adapter for Illumina (red tube) in 10 mM Tris-HCl, pH 8.0 with 10 mM NaCl as indicated below.

Input DNA in the End Prep reaction	Adapter dilution (volume of adapter: total volume)	Working adapter concentration
250 ng - 101 ng	No dilution	15 uM
100 ng – 5 ng	10-fold (1:10)	1.5 uM
Less than 5 ng	25-fold (1:25)	0.6 uM

2.2. Add the following components directly to the FS reaction mixture from 1.1(35 uL):

Component	Volume (uL)
NEBNext Ultra II Ligation Master Mix (red tube)	30
NEBNext Ultra II Ligation enhancer (green tube)	1
NEBNext adapter for Illumina	2.5

Notes:
- Mix the Ultra II Ligation Master Mix by pipetting up and down several times prior to adding to the reaction.
- The Ligation master mix and ligation enhancer can be mixed ahead of time and is stable for at least 8 hours at 4°C. Do not premix the adapter prior to use in the adapter ligation step.
- The NEBNext adapter is provided in NEBNext Multiplex Oligos for Illumina (96 Unique Dual Index Primer Pairs)

2.3. Set a pipette to 50 uL and pipette entire volume up and down at least 10 times to mix thoroughly. Perform a quick spin to collect all liquid from the sides of the tube.
Note: The NEBNext Ultra II Ligation master mix is very viscous. Care should be taken to ensure adequate mixing of the ligation reaction as incomplete mixing will result in reduced ligation efficiency. The presence of a small amount of bubbles will not interfere with performance.

2.4. Incubate at 20°C for 15 minutes in a thermocycler with the heated lid off.

2.5. Add 3 uL of USER enzyme (red tube) to the ligation mixture.
Note: This step is only required for use with NEBNext adapters. USER enzyme is provided in NEBNext Multiplex Oligos for Illumina (96 Unique Dual Index Primer Pairs)

2.6. Mix well and incubate at 37°C for 15 minutes in a thermocycler with the heated lid set to ≥47°C

2.7. Add 57uL (0.8X) re-suspended AMPure XP beads to the ligation reaction (87uL).

2.8. Follow steps in the AMPure XP bead clean-up section (Appendix E).

2.9. Elute the DNA target from the beads by adding 17 uL of 10mM Tris-HCl or 0.1X TE.

2.10. Transfer 15 uL to a new PCR tube for amplification.

3. PCR enrichment of Adapter-Ligated DNA

3.1. Add the following components to a sterile strip tube:

Component	Volume (uL)
Adapter ligated DNA fragments (from above)	15
Unique dual index primer pair*	10
NEBNext Ultra II Q5 master mix (blue tube)	25
Total volume	50

*The primers are provided in NEBNext® Multiplex Oligos for Illumina® (96 Unique Dual Index Primer Pairs). Please refer to the NEB #E6440 manual for valid barcode combination and tips for setting up PCR reactions

3.2. Set a pipette to 40 uL and then pipette the entire volume up and down at least 10 times to mix thoroughly. Perform a quick spin to collect all liquid from the sides of the tube.

3.3. Place tube on a thermocycler and perform PCR amplification using the following PCR cycling conditions:

Cycle step	Temperature	Time	# of cycles
Initial denaturation	98°C	30 seconds	1
Denaturation	98°C	10 seconds	3-15*
Annealing/extension	65°C	75 seconds	
Final extension	65°C	5 minutes	1
Hold	4°C	∞	

*Follow the recommendations for cycle number listed in the table below.

Cycle recommendations

Input DNA in the end prep reaction	# of cycles required to generate a library yield of:	
	100 ng	1 ug
250 ng	2-3	3-4
100 ng	3-4	4-5
50 ng	4-5	5-6
10 ng	6-7	8-9
5 ng	7-8	9-10
1 ng	8-10	11-12
0.5 ng	9-10	12-13
0.1ng	12-13	N/A

3.4. Add 0.9X AMPure XP beads to the PCR reactions (45uL).

3.5. Follow steps in the AMPure XP bead clean-up section (Appendix E).

3.6. Elute DNA target from beads into 33 uL 0.1X TE.

3.7. Transfer 30 uL supernatant to a new PCR tube. Libraries can be store at -20C°.

3.8. Check size distribution of libraries and quantitate library concentration.

4. Sizing and quantitation

4.1. Allow TapeStation reagents to equilibrate at room temperature for 30 minutes prior to use.

4.2. Vortex reagents well before use.

4.3. To prepare ladder, mix 2 uL high sensitivity D1000 sample buffer with 2 uL high sensitivity D1000 ladder.

4.4. To prepare sample, mix 2 uL high sensitivity D1000 sample buffer with 2 uL sample.
4.5. Spin down, then vortex using IKA vortexer and adapter at 2000 rpm for 1 minute.
4.6. Spin down to position the sample at the bottom of the tube.
4.7. Load samples into the 2200 TapeStation instrument and follow the software procedure for analysis.
4.8. Quantitate 1 uL library sample with Qubit dsDNA HS kit (Appendix F).

MiSeq sequencing

Protocol Notes

This procedure requires Illumina-style libraries that have been quality-controlled and quantitated using the recommended procedures (i.e. TapeStation and Qubit or qPCR). Exact loading concentrations may vary by machine or lab-dependent factors. For more details on loading and running the MiSeq, consult the more detailed manuals at http://www.illumina.com.

Required Reagents

Company	Product	Catalog number
Illumina	MiSeq reagent kit v3	MS-102-3003
Illumina	PhiX control kit v3	FC-110-3001
	NaOH	
	Nuclease-free water	

Procedure

1. Dilute and Pool Libraries

1.1. Calculate the molar concentration of each library to be diluted using average size from the TapeStation and mass from Qubit, using the following equation:

$$\frac{concentration\ (ng/uL)}{660g/mol \times avg\ library\ fragment\ size} \times 10^6\ uL/L = concentration\ (nM)$$

1.2.

1.3. Make a 4nM dilution of each library.

1.4. Combine equal volumes of each diluted library into a new tube. This is the 4nM library pool.

2. Denature Libraries

2.1. Make a fresh dilution of 0.2N NaOH by combining the following volumes in a microcentrifuge tube:

800 uL laboratory-grade water
200 stock 1.0N NaOH

2.2. Remove HT1 from freezer and thaw at room temperature. Store at 2°C to 8°C until you are ready to dilute denatured libraries.

2.3. Combine the following volumes in a microcentrifuge tube:

5 uL 4nM library
5 uL 0.2N NaOH

2.4. Vortex briefly and then centrifuge at 280 x g for 1 minute.

2.5. Incubate at room temperature for 5 minutes.

2.6. Add 990 uL pre-chilled HT1 to the tube containing denatured library. The result is 1 mL of a 20pM denatured library.

2.7. Dilute the 20pM library to the desired concentration, see table below:

Concentration	6 pM	8 pM	10 pM	12 pM	15 pM	20 pM
20 pM library	180 uL	240 uL	300 uL	360 uL	450 uL	600 uL
Pre-chilled HT1	420 uL	360 uL	300 uL	240 uL	150 uL	0 uL

2.8. Invert to mix and then pulse centrifuge.

2.9. Dilute stock PhiX to 4nM by combining:
2 uL 10 nM PhiX library
3 uL 10 mM Tris-Cl, pH 8.5 with 0.1% Tween 20

2.10. Denature the PhiX control by adding the following volumes in a microcentrifuge tube:
5 uL 4nM PhiX library
5 uL 0.2N NaOH
Remaining 4nM PhiX can be frozen and reused

2.11. Vortex briefly to mix and centrifuge at 280 x g for 1 minute.

2.12. Incubate at room temperature for 5 minutes.

2.13. Dilute denatured PhiX library to 20 pM by adding 990 uL pre-chilled HT1 to the PhiX tube. Invert to mix.

2.14. If using a MiSeq reagent kit v2, dilute 20 pM PhiX library to 12.5 pM by adding the following volumes in a microcentrifuge tube:
375 uL 20pM denatured PhiX library
225 uL pre-chilled HT1

2.15. Combine library and PhiX control according to the table below:

Denatured and diluted PhiX	30 uL
Denatured and diluted library	570 uL

2.16. Set aside on ice until you are ready to load it onto the reagent cartridge.

3. Load and Run MiSeq

3.1. Thaw frozen reagents overnight at 4°C overnight or in a RT water bath.

3.2. Mix reagents thoroughly by inverting several times. Inspect the bottom of reagent cartridge to ensure all liquids return to the bottom of each tube without any air bubbles.

3.3. Using a 1000 uL pipette tip, piece the foil on position 17.

3.4. Using a fresh 1000 uL pipette tip, transfer the denatured and library (with PhiX spiked) into position 17.

3.5. Generate Sample Sheet using MiSeq Experiment Manager.

3.6. Load MiSeq according to onscreen instructions in the MiSeq Control software.

4. Generation of consensus sequences from MiSeq data

The Illumina MiSeq provides very high-quality data, and consensus sequenced may be generated by variety of methods, including commercial tools such as Geneious and CLC Genomics Workbench. The procedure outlined here is a suggestion using free, open source tools.

Software	Source URL
cutadapt 2.3+	https://github.com/marcelm/cutadapt
bowtie2	https://github.com/BenLangmead/bowtie2
seqtk	https://github.com/lh3/seqtk
SAMtools 1.9	http://www.htslib.org/
BCFtools 1.9	http://www.htslib.org/
IGV	http://software.broadinstitute.org/software/igv/

4.1. Trim reads for quality (Q25+) and for adapters on both ends. Then trim primer sequences (a hard 30 bases on each end), keeping only sequenced that are at least 75 bases. For reads <150 bases, this will need to be modified.

```
cutadapt -j $threads -g GTTTCCCAGTCACGATA -G GTTTCCCAGTCACGATA  \
   -a TATCGTGACTGGGAAAC -A TATCGTGACTGGGAAAC -g ACACTCTTTCCCTACACGACGCTCTTCCGATCT \
   -G ACACTCTTTCCCTACACGACGCTCTTCCGATCT -a AGATCGGAAGAGCACACGTCTGAACTCCAGTCA \
   -A AGATCGGAAGAGCGTCGTGTAGGGAAAGAGTGT -n 3  -m 75   -q 25  \
   --interleaved $read1 $read2 | cutadapt -j $threads --interleaved -m 75 -u 30 \
   -u -30 -U 30 -U -30 -o $read1.trim.fastq -p $read2.trim.fastq -
```

4.2. Map reads to reference sequence

```
bowtie2-build MN908947.fasta MN908947
bowtie2 --sensitive-local  -p $threads -x MN908947 \
   -1 $read1.trim.fastq -2 $read2.trim.fastq -S $samfile
samtools view -b $samfile | samtools sort - -o $bamfile
samtools index $bamfile
```

4.3. Call variants, generate consensus sequence. This will call positions covered by at least 100 reads.

```
samtools mpileup -aa -d 8000 -uf MN908947.fasta $bamfile |\
   bcftools call -Mc |tee -a $vcf | \
   vcfutils.pl vcf2fq -d 100 -D 100000000|\
   seqtk seq -A - | sed  '2~2s/[actg]/N/g'  > $consensusfasta
```

Appendix A – Singleplex PCR Primers

Amplicon	1st round	Sequence	Size	2nd round	Sequence	Size
PCR1	1F_209_1	GTTGCAGCCGATCATCAGCAC	756	W1_2L_368	TGGAGGAGGTCTTATCAGAGGC	597
	SC2M1-2_RIGHT_965	GTTCACGGCAGCAGTATACACC		SC2M1-2_RIGHT_965	GTTCACGGCAGCAGTATACACC	
PCR2	W1_2F_00826_1	AACAACTTCTGTGGCCCTGATG	904	W1_2F_00850_2	TACCCTCTTGAGTGCATTAAAG	853
	W1_2R_01730_1	TCCACAAAAGCACTTGTGGAAGC		W1_2R_01703_2	AAAGATGCCAAAATAATGGCG	
PCR3	W1_3F_01573_1	GGTGTTGTTGGAGAAGGTTCCG	938	W1_3F_01596_2	AGGTCTTAATGACAACCTTCTTG	894
	W1_3R_02511_1	TGTGGGAAGTGTTTCTCCCTC		W1_3R_02490_2	TAAGAAGATAATTTCTTTTGGG	
PCR4	W1_4F_02387_1	CATTTGTCACGCACTCAAAGGG	942	W1_4F_02404_2	AAGGGATTGTACAGAAAGTGTG	903
	W1_4R_03329_1	GTCTGAACAACTGGTGTAAGTTCC		W1_4R_03307_2	CCATCTCTAATTGAGGTTGAAC	
PCR5	W1_5F_03185_1	AGCAAGAAGAAGATTGGTTAGATGATG	1014	W1_5F_03208_2	GATGATAGTCAACAAACTGTTGG	868
	W1_5R_04199_1	ATTTCAGTAGTGCCACCAGCC		W1_5R_04175_2	TTAGTAGGTATAACCACAGCAG	
PCR6	W1_6F_04054_1	CATCCAGATTCTGCCACTCTTG	968	W1_6F_04073_2	TTGTTAGTGACATTGACATCAC	932
	W1_6R_05022_1	CATGTCCACAACTTGCGTGTG		W1_6R_05005_2	TGTGGAGGTTAATGTTGTCTAC	
PCR7	W1_7F_04884_1	TCCTACCACATTCCACCTAGATGG	933	W1_7F_04904_2	ATGGTGAAGTTATCACCTTTG	893
	W1_7R_05817_1	AGCACCGTCTATGCAATACAAAG		W1_7R_05797_2	AAGTTTCTTTAGAAGTTATATG	
PCR8	W1_8F_05676_1	TGTTATGATGTCAGCACCACCTG	977	W1_8F_05699_2	CTCAGTATGAACTTAAGCATGG	933
	W1_8R_06653_1	ACAGCAGCTAAACCATGAGTAGC		W1_8R_06632_2	GCAAGGGTTTTCAAACCTAATAC	
PCR9	W1_9F_06522_1	TACAGAAGAGGTTGGCCACAC	954	W1_9F_06543_2	AGATCTAATGGCTGCTTATGTAG	919
	W1_9R_07476_1	ACAACCGTCTACAACATGCAC		W1_9R_07462_2	CATGCACATAACTTTTCCATAC	
PCR10	W1_10F_07326_1	TGCAGTACATTTTATTAGTAATTCTTGG	999	W1_10F_07356_2	TATGTGGTTAATAATTAATCTTG	952
	W1_10R_08325_1	GTCACGGGGTGTCATGTTTTC		W1_10R_08308_2	TTTCAACTTTGTTATAGGTGAGC	
PCR11	W1_11F_08170_1	GCAGCTCGGCAAGGGTTTGTTG	952	W1_11F_08184_2	GTTTGTTGATTCAGATGTAGAAAC	922
	W1_11R_09122_1	CGTGTGTCAGGGCGTAAACTTTC		W1_11R_09106_2	AACTTTCATAAGCAACAGAACC	
PCR12	W1_12F_08996_1	CAGCTTGTGTTTTGGCTGCTG	980	W1_12F_09017_2	AATGTACAATTTTTAAAGATGC	949
	W1_12R_09976_1	GAGCCTTTGCGAGATGACAAC		W1_12R_09966_2	GAGATGACAACAAGCAGCTTC	
PCR13	W1_13F_09831_1	GTATCTAAAGTTGCGTAGTGATG	1005	W1_13F_09850_2	GATGTGCTATTACCTCTTACGC	966
	W1_13R_10836_1	AACGGCAATTCCAGTTTGAGC		W1_13R_10816_2	CAGAAAGAGGTCCTAGTATGTC	
PCR14	W1_14F_10686_1	TGTTATAAATGGAGACAGGTGG	984	W1_14F_10708_2	TTTCTCAATCGATTTACCACAAC	949
	W1_14R_11670_1	GCGGTTGAGTAAACAAAAGAGGC		W1_14R_11657_2	CAAAAGAGGCCAAAGTAACAAG	
PCR15	W1_15F_11527_1	GCCAGAGGTATTGTTTTTATGTGTGT	911	W1_15F_11527_2	GCCAGAGGTATTGTTTTTATGTGTGT	889
	W1_15R_12438_1	GGGAACACAACCATCTCTTGC		W1_15R_12416_2	TTGTTGATAATGTTGTTGAGTGC	
PCR16	W1_16F_12311_1	CTAGATCTGAGGACAAGAGGGC	929	W1_16F_12327_2	GAGGGCAAAAGTTACTAGTGC	892
	W1_16R_13240_1	ACGATGCACCACCAAAGGATTC		W1_16R_13219_2	CTTGATCCATATTGGCTTCCGG	
PCR17	W1_17F_13112_1	ATCTAGCTAGTGGGGGACAACC	930	W1_17F_13126_2	GGACAACCAATCACTAATTGTG	902
	W1_17R_14042_1	AATACCAGCATTTCGCATGGCA		W1_17R_14028_2	GCATGGCATCACAGAATTGTAC	
PCR18	W1_18F_13873_1	TACTTGTCACATACAATTGTTGTGATG	914	W1_18F_13873_1	TACTTGTCACATACAATTGTTGTGATG	914
	18R_14809_1	GATAGTAGTCATAATCGCTGATAGCAG		W1_18R_14787_1	TAGCAGCATTACCATCCTGAGC	
PCR19	W1_19F_14655_1	GCTTTTCAAACTGTCAAACCCGG	902	W1_19F_14670_2	AAACCCGGTAATTTTAACAAAG	879
	W1_19R_15557_1	TGCATTAACATTGGCCGTGAC		W1_19R_15549_2	CATTGGCCGTGACAGCTTGAC	
PCR20	W1_20F_15429_1	AGTGAAATGGTCATGTGTGGCG	971	W1_20F_15441_2	ATGTGTGGCGGTTCACTATATG	939
	W1_20R_16400_1	ACAACCTGGAGCATTGCAAAC		W1_20R_16380_2	CATACGGATTAACAGACAAGAC	
PCR21	21F_16221_1	GCATACAGTCTTACAGGCTGTTGG	919	W1_21F_16291_2	GCATACGTAGACCATTCTTATG	849
	21R_17140_1	CAGAAGGGTAGTAGAGAGCTAGGC		21R_17140_1	CAGAAGGGTAGTAGAGAGCTAGGC	
PCR22	W1_22F_17065_1	ATTCTACACTCCAGGGACCACC	970	W1_22F_17082_2	CCACCTGGTACTGGTAAGAGTC	930
	W1_22R_18035_1	TAAAGTTGCCACATTCCTACGTGG		W1_22R_18012_2	GAATTTCAAGACTTGTAAATTG	
PCR23	W1_23F_17881_1	CCACTGAAACAGCTCACTCTTG	1019	W1_23F_17901_2	TGTAATGTAAACAGATTTAATG	978
	W1_23R_18900_1	TAACAAAGCACTCGTGGACAGC		W1_23R_18879_2	CTAGACACCTAGTCATGATTGC	
PCR24	W1_24F_18767_1	TGTTCAACAATGGGGTTTTACAGG	910	W1_24F_18786_2	ACAGGTAACCTACAAAGCAACC	879
	W1_24R_19677_1	CCTGTTGTCCATCAAAGTGTCCC		W1_24R_19665_2	CAAAGTGTCCCTTATTTACAAC	

PCR25	W1_25F_19546_1	CAGCTGGCTTTAGCTTGTGGG	936	W1_25F_19546_1	CAGCTGGCTTTAGCTTGTGGG	936
	25R_20572_1	CAACCTTAGAAACTACAGATAAATCTTG		W1_25R_20482_1	GATGAACCTGTTTGCGCATCTG	
PCR26	W1_26F_20343_1	CATAGTCAGTTAGGTGGTTTAC	972	W1_26F_20356_2	GTGGTTTACATCTACTGATTGG	944
	W1_26R_21315_1	CTATTTGTTCGCGTGGTTTGCC		W1_26R_21300_2	GTTTGCCAAGATAATTACATCC	
PCR27	27F_21136_1	AAGCTAGCTCTTGGAGGTTCCG	926	W1_27F_21204_2	CTCATGGGACACTTCGCATGGTGG	895
	27R_22218_1	CCCTGAGGGAGATCACGCAC		W1_27R_22099_2	CAAGGTCCATAAGAAAAGGCTG	
PCR28	W1_28F_21976_1	CCATTTTTGGGTGTTTATTACC	1017	W1_28F_21996_2	CCACAAAAACAACAAAAGTTGG	979
	W1_28R_22993_1	TGCTACCGGCCTGATAGATTTC		W1_28R_22975_2	TTTCAGTTGAAATATCTCTCTC	
PCR29	W1_29F_22847_1	TTACAGGCTGCGTTATAGCTTGG	965	W1_29F_22864_2	GCTTGGAATTCTAACAATCTTG	931
	W1_29R_23812_1	TGCTGCATTCAGTTGAATCACC		W1_29R_23795_2	TCACCACAAATGTACATTGTAC	
PCR30	W1_30F_23681_1	ACTCTAATAACTCTATTGCCATACCCAC	944	W1_30F_23704_2	CCCACAAATTTTACTATTAGTG	906
	W1_30R_24625_1	CAGAAGCTCTGATTTCTGCAGC		W1_30R_24610_2	CTGCAGCTCTAATTAATTGTTG	
PCR31	W1_31F_24492_1	AAATGATATCCTTTCACGTCTTGACAAAG	999	W1_31F_24514_2	GACAAAGTTGAGGCTGAAGTGC	962
	W1_31R_25491_1	TTGCAGTAGCGCGAACAAAATC		W1_31R_25476_2	CAAAATCTGAAGGAGTAGCATC	
PCR32	W1_32F_25348_1	CCAGTGCTCAAAGGAGTCAAATTAC	1019	W1_32F_25357_2	AAAGGAGTCAAATTACATTACAC	1001
	W1_32R_26367_1	ACGCACACAATCGAAGCGCAG		W1_32R_26358_2	ATCGAAGCGCAGTAAGGATGGC	
PCR33	W1_33F_26222_1	ACAAGCTGATGAGTACGAACTTATG	906	W1_33F_26241_2	CTTATGTACTCATTCGTTTCGG	874
	W1_33R_27128_1	TGCCAATCCTGTAGCGACTGTATGC		W1_33R_27115_2	GCGACTGTATGCAGCAAAACC	
PCR34	W1_34F_26988_1	TAGGACGCTGTGACATCAAGG	1018	W1_34F_26999_2	GACATCAAGGACCTGCCTAAAG	993
	W1_34R_28006_1	AGGACACGGGTCATCAACTAC		W1_34R_27992_2	CAACTACATATGGTTGATGTTG	
PCR35	35F_27834_1	ATCTTTTGGTTCTCACTTGAACTGC	1021	W1_35F_27875_1	TGTCACGCCTAAACGAACATG	980
	35th_R2_28855	TGAACTGTTGCGACTACGTGATG		35th_R2_28855	TGAACTGTTGCGACTACGTGATG	
PCR36	W1_36F_28694_1	CACCAAAAGATCACATTGGCAC	1030	W1_36F_28716_2	CCGCAATCCTGCTAACAATGC	1008
	W1_36R_29724_1	TGTGGTGGCTCTTTCAAGTCC		W1_36R_29724_2	TGTGGTGGCTCTTTCAAGTCC	
PCR37	W1_37F_29551_1	AGGCAGATGGGCTATATAAACG	322	W1_37F_29596_2	TATAGTCTACTCTTGTGCAGAATG	280
	W1_37R_29873_2	TTTTGTCATTCTCCTAAGAAGC		W1_37R_29873_2	TTTTGTCATTCTCCTAAGAAGC	
PCR38	SC2M1-1_LEFT2_1	TTAAAGGTTTATACCTTCCCAGG	495	0_1b	TTAAAGGTTTATACCTTCCCAGGTA	490
	SC2M1-1_RIGHT2_495	CGAGCATCCGAACGTTTGATGA		W1_1R_490	CATCCGAACGTTTGATGAACAC	

Appendix B – Sequencing Primers

Sequencing primer to amplicon matrix

PCR Product	Sequencing primers			
PCR1	W1_2L_368	SC2M1-2_RIGHT_965	SC2M1-2_LEFT_445	SC2M1-1_RIGHT_574
PCR2	W1_2F_00850_2	W1_2R_01703_2	W1_4F_1067*	W1_3R_1206*
PCR3	W1_3F_01596_2	W1_3R_02490_2	W1_6L_1819	W1_5R_1969
PCR4	W1_4F_02404_2	W1_4R_03307_2	W1_9L_2948*	W1_8R_3094*
PCR5	W1_5F_03208_2	W1_5R_04175_2	W1_11L_3638*	W1_10R_3792*
PCR6	W1_6F_04073_2	W1_6R_05005_2	W1_13L_4307*	W1_12R_4522*
PCR7	W1_7F_04904_2	W1_7R_05797_2	W1_15L_5159*	W1_14R_5299*
PCR8	W1_8F_05699_2	W1_8R_06632_2	SC2M1-16_LEFT_6030	SC2M1-15_RIGHT_6172
PCR9	W1_9F_06543_2	W1_9R_07462_2	W1_20L_6877*	W1_19R_7009*
PCR10	W1_10F_07356_2	W1_10R_08308_2	W1_22L_7625*	W1_21R_7771*
PCR11	W1_11F_08184_2	W1_11R_09106_2	W1_25L_8669*	W1_24R_8794*
PCR12	W1_12F_09017_2	W1_12R_09966_2	W1_27L_9308*	W1_26R_9459
PCR13	W1_13F_09850_2	W1_13R_10816_2	W1_29R_10593*	W1_30L_10448*
PCR14	W1_14F_10708_2	W1_14R_11657_2	W1_32L_11111*	W1_31R_11251*
PCR15	W1_15F_11527_2	W1_15R_12416_2	W1_34L_11808*	W1_33R_11948*
PCR16	W1_16F_12327_2	W1_16R_13219_2	W1_37L_12878*	W1_35R_12700*
PCR17	W1_17F_13126_2	W1_17R_14028_2	W1_39L_13600*	W1_38R_13741*
PCR18	W1_18F_13873_1	W1_18R_14787_1	W1_41L_14342*	W1_40R_14503
PCR19	W1_19F_14670_2	W1_19R_15549_2	W1_43L_14960*	W1_42R_15108*
PCR20	W1_20F_15441_2	W1_20R_16380_2	W1_46L_16004*	W1_44R_15773*
PCR21	W1_21F_16291_2	21R_17140_1	W1_48L_16735*	W1_46R_16490*
PCR22	W1_22F_17082_2	W1_22R_18012_2	W1_50L_17424*	W1_49R_17553*
PCR23	W1_23F_17901_2	W1_23R_18879_2	W1_53L_18503*	W1_52R_18667*
PCR24	W1_24F_18786_2	W1_24R_19665_2	W1_55L_19277*	W1_54R_19405*
PCR25	W1_25F_19546_1	W1_25R_20482_1	W1_57L_20013*	W1_56R_20146*
PCR26	W1_26F_20356_2	W1_26R_21300_2	W1_59L_20656*	W1_58R_20796*
PCR27	W1_27F_21204_2	W1_27R_22099_2	W1_61L_21411*	W1_60R_21562
PCR28	W1_28F_21996_2	W1_28R_22975_2	W1_64L_22457	W1_63R_22612*
PCR29	W1_29F_22864_2	W1_29R_23795_2	W1_66L_23182*	W1_65R_23308*
PCR30	W1_30F_23704_2	W1_30R_24610_2	W1_69L_24259*	W1_67R_24002*
PCR31	W1_31F_24514_2	W1_31R_25476_2	W1_71L_24935*	W1_70R_25075*
PCR32	W1_32F_25357_2	W1_32R_26358_2	SC2M1-66_LEFT_25665	SC2M1-65_RIGHT_25790
PCR33	W1_33F_26241_2	W1_33R_27115_2	SC2M1-68_LEFT_26454	SC2M1-67_RIGHT_26590
PCR34	W1_34F_26999_2	W1_34R_27992_2	SC2M1-71_LEFT_27650	SC2M1-69_RIGHT_27432
PCR35	W1_35F_27875_1	35th R2_28855	W1_81L_28414*	SC2M1-71_RIGHT_28203
PCR36	W1_36F_28716_2	W1_36R_29724_2	SC2M1-75_LEFT_29344	SC2M1-74_RIGHT_29469
PCR37	W1_37F_29596_2	W1_37R_29873_2		
PCR38	0_1b	W1_1R_490		

*Primer sequence in table below.

SC2M1 primers are sourced from the multiplex primer set (Appendix D). Others are primers from Appendix A.

Additional Sequencing Primer Sequences

Name	Sequence		Nmae	Sequence
W1_4F_1067	GGGAATGTCCAAATTTTGTATTTCC		W1_41L_14342	TTTGGATGACAGATGCATTCTGC
W1_3R_1206	TGGTTGCATTCATTTGGTGACG		W1_43L_14960	TAAATGGGGTAAGGCTAGACTTTATTATG
W1_9L_2948	TTGATTTAGATGAGTGGAGTATGGCTAC		W1_42R_15108	CGGTGCGAGCTCTATTCTTTGC
W1_8R_3094	ATGGCTCAAACTCTTCTTCTTCAC		W1_46L_16004	GATTGAACGGTTCGTGTCTTTAGC
W1_11L_3638	GTGAAGACATTCAACTTCTTAAGAGTGC		W1_44R_15773	GCTAGCCACTAGACCTTGAGATGC
W1_10R_3792	AGCTAAGTAGACATTTGTGCGAAC		W1_48L_16735	GGGAAGTTGGTAAACCTAGACCAC
W1_13L_4307	GAACTGTTTCTTGGAATTTGCGAG		W1_46R_16490	AGCACACAATGGAAAACTAATGGG
W1_12R_4522	CACCATAATCAACCACACCCTC		W1_50L_17424	GTGTACATTGGCGACCCTGCTC
W1_15L_5159	TTGAGTACTACCACACAACTGATCC		W1_49R_17553	TTCCGAGGAACATGTCTGGACC
W1_14R_5299	CAGTGGCAAGATAACAGTTGTTATC		W1_53L_18503	AGGACTTCCTTGGAATGTAGTGCG
W1_20L_6877	AGTGTCGGTAAATTTTGTCTAGAGGC		W1_52R_18667	CATAGACAACAGGTGCGCTCAG
W1_19R_7009	CAGCGGTTGAGTAGATTAAAGAACC		W1_55L_19277	TTGTGATGGTGGCAGTTTGTATG
W1_22L_7625	ATTGTGATACATTCTGTGCTGGTAGTAC		W1_54R_19405	CCATGAGACTCACATGGACTGTC
W1_21R_7771	GATGGATGGAACCATTCTTCACTG		W1_57L_20013	GACTTATTTAGAAATGCCCGTAATGGTG
W1_25L_8669	TTTCAAGTGAAATCATAGGATACAAGG		W1_56R_20146	AACTGTGTTTTTACGGCTTCTCC
W1_24R_8794	TACCACCACGCTGGCTAAACC		W1_59L_20656	AATCTAGTCAAGCGTGGCAACC
W1_27L_9308	CAGGAGTTTTCTGTGGTGTAGATGC		W1_58R_20796	ATTTTGCGACATTCATCATTATGCC
W1_29R_10593	GTTACCTTCTAAGTCTGTGCCAGC		W1_61L_21411	CTTAAATTAAGGGGTACTGCTGTTATG
W1_30L_10448	CCAATTTCACTATTAAGGGTTCATTCC		W1_63R_22612	AAACAGATGCAAATCTGGTGGC
W1_32L_11111	TGGGTATTATTGCTATGTCTGCTTTTG		W1_66L_23182	TTCAACTTCAATGGTTTAACAGGCAC
W1_31R_11251	TACGCATCACCCAACTAGCAGG		W1_65R_23308	CAAGTGTCTGTGGATCACGGAC
W1_34L_11808	TGGCAAACCTTGTATCAAAGTAGC		W1_69L_24259	ATGCAAATGGCTTATAGGTTTAATGG
W1_33R_11948	TGTAACTGGACACATTGAGCCC		W1_67R_24002	TTGCTTGGTTTTGATGGATCTGG
W1_37L_12878	TCTATACAGAACTGGAACCACCTTG		W1_71L_24935	ACTGTGATGTTGTAATAGGAATTGTCAAC
W1_35R_12700	AGACATCTGTCGTAGTGCAACAGG		W1_70R_25075	TGCCAGAGATGTCACCTAAATCAAC
W1_39L_13600	GTCGCTTCCAAGAAAAGGACG		W1_81L_28414	AATACTGCGTCTTGGTTCACCG
W1_38R_13741	AAGTCATGTTTAGCAACAGCTGG			

Appendix C – Plate Setup for Nested PCR and Sanger Sequencing

Primers are added to each PCR reaction (PCR1-PCR38) prior to adding RNA. The layout stays the same until sequencing reactions are run.

We recommend making PCR primer plates (R1 and R2) in the same format so that primers may be added by multichannel pipetting.

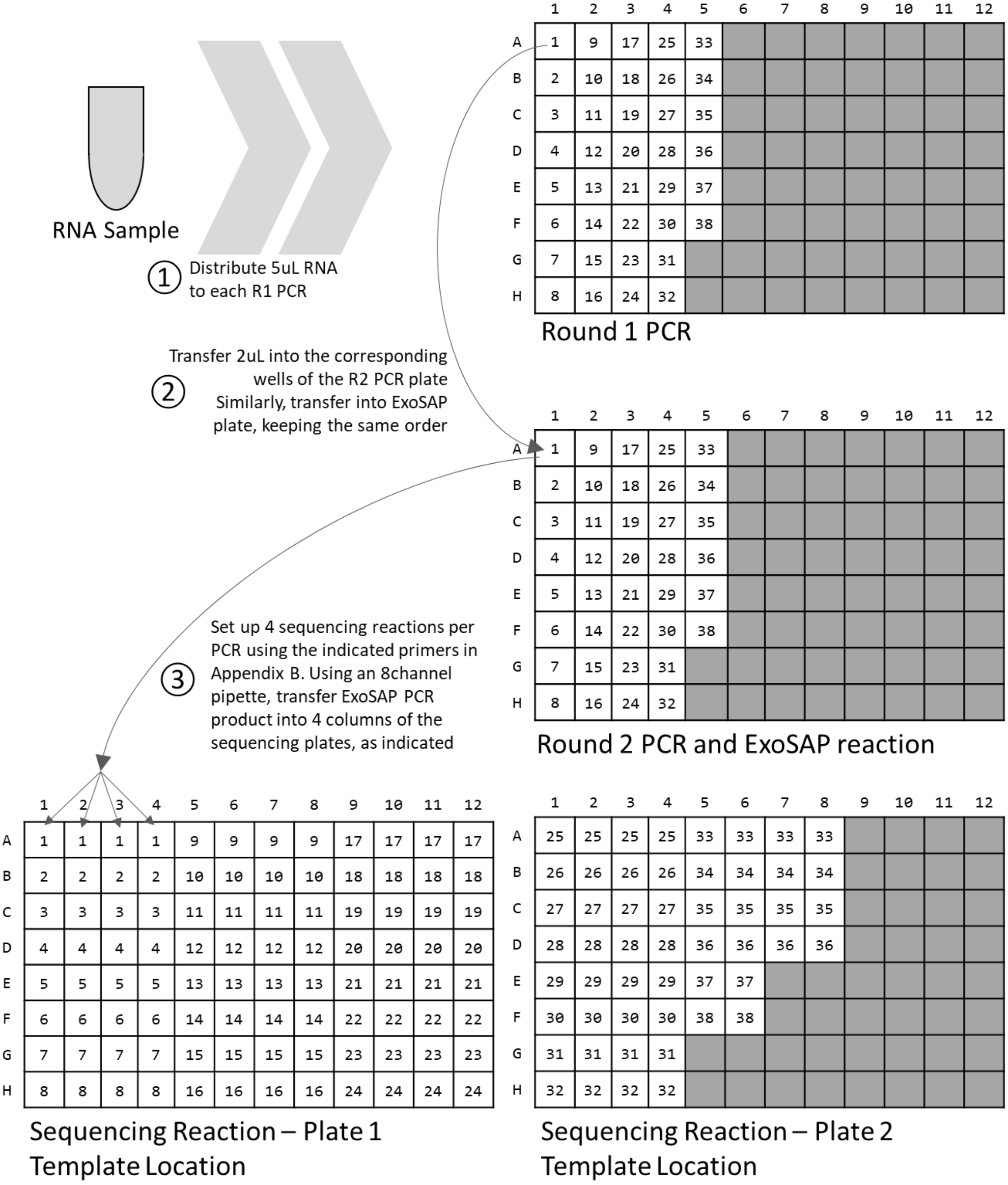

Round 1 PCR

	1	2	3	4	5	6	7	8	9	10	11	12
A	1	9	17	25	33							
B	2	10	18	26	34							
C	3	11	19	27	35							
D	4	12	20	28	36							
E	5	13	21	29	37							
F	6	14	22	30	38							
G	7	15	23	31								
H	8	16	24	32								

Round 2 PCR and ExoSAP reaction

	1	2	3	4	5	6	7	8	9	10	11	12
A	1	9	17	25	33							
B	2	10	18	26	34							
C	3	11	19	27	35							
D	4	12	20	28	36							
E	5	13	21	29	37							
F	6	14	22	30	38							
G	7	15	23	31								
H	8	16	24	32								

	1	2	3	4	5	6	7	8	9	10	11	12
A	1	1	1	1	9	9	9	9	17	17	17	17
B	2	2	2	2	10	10	10	10	18	18	18	18
C	3	3	3	3	11	11	11	11	19	19	19	19
D	4	4	4	4	12	12	12	12	20	20	20	20
E	5	5	5	5	13	13	13	13	21	21	21	21
F	6	6	6	6	14	14	14	14	22	22	22	22
G	7	7	7	7	15	15	15	15	23	23	23	23
H	8	8	8	8	16	16	16	16	24	24	24	24

Sequencing Reaction – Plate 1
Template Location

	1	2	3	4	5	6	7	8	9	10	11	12
A	25	25	25	25	33	33	33	33				
B	26	26	26	26	34	34	34	34				
C	27	27	27	27	35	35	35	35				
D	28	28	28	28	36	36	36	36				
E	29	29	29	29	37	37						
F	30	30	30	30	38	38						
G	31	31	31	31								
H	32	32	32	32								

Sequencing Reaction – Plate 2
Template Location

We recommend making sequencing primer plates as shown below, so that primers may be rapidly added to the sequencing reactions. Primer sequences may be found in Appendices A and D.

W1_2L_368	SC2M1-2_RIGHT_965	SC2M1-2_LEFT_445	SC2M1-1_RIGHT_574	W1_9F_06543_2	W1_9R_07462_2	W1_20L_6877	W1_19R_7009	W1_17F_13126_2	W1_17R_14028_2	W1_39L_13600	W1_38R_13741
W1_2F_00850_2	W1_2R_01703_2	W1_4F_1067	W1_3R_1206	W1_10F_07356_2	W1_10R_08308_2	W1_22L_7625	W1_21R_7771	W1_18F_13873_1	W1_18R_14787_1	W1_41L_14342	W1_40R_14503
W1_3F_01596_2	W1_3R_02490_2	W1_6L_1819	W1_5R_1969	W1_11F_08184_2	W1_11R_09106_2	W1_25L_8669	W1_24R_8794	W1_19F_14670_2	W1_19R_15549_2	W1_43L_14960	W1_42R_15108
W1_4F_02404_2	W1_4R_03307_2	W1_9L_2948	W1_8R_3094	W1_12F_09017_2	W1_12R_09966_2	W1_27L_9308	W1_26R_9459	W1_20F_15441_2	W1_20R_16380_2	W1_46L_16004	W1_44R_15773
W1_5F_03208_2	W1_5R_04175_2	W1_11L_3638	W1_10R_3792	W1_13F_09850_2	W1_13R_10816_2	W1_29R_10593	W1_30L_10448	W1_21F_16291_2	21R_17140_1	W1_48L_16735	W1_46R_16490
W1_6F_04073_2	W1_6R_05005_2	W1_13L_4307	W1_12R_4522	W1_14F_10708_2	W1_14R_11657_2	W1_32L_11111	W1_31R_11251	W1_22F_17082_2	W1_22R_18012_2	W1_50L_17424	W1_49R_17553
W1_7F_04904_2	W1_7R_05797_2	W1_15L_5159	W1_14R_5299	W1_15F_11527_2	W1_15R_12416_2	W1_34L_11808	W1_33R_11948	W1_23F_17901_2	W1_23R_18879_2	W1_53L_18503	W1_52R_18667
W1_8F_05699_2	W1_8R_06632_2	SC2M1-16_LEFT_6030	SC2M1-15_RIGHT_6172	W1_16F_12327_2	W1_16R_13219_2	W1_37L_12878	W1_35R_12700	W1_24F_18786_2	W1_24R_19665_2	W1_55L_19277	W1_54R_19405

Sequencing Reaction – Plate 1
Sequencing Primer Location

W1_25F_19546_1	W1_25R_20482_1	W1_57L_20013	W1_56R_20146	W1_33F_26241_2	W1_33R_27115_2	SC2M1-68_LEFT_26454	SC2M1-67_RIGHT_26590				
W1_26F_20356_2	W1_26R_21300_2	W1_59L_20656	W1_58R_20796	W1_34F_26999_2	W1_34R_27992_2	SC2M1-71_LEFT_27650	SC2M1-69_RIGHT_27432				
W1_27F_21204_2	W1_27R_22099_2	W1_61L_21411	W1_60R_21562	W1_35F_27875_1	35th R2_28855	W1_81L_28414	SC2M1-71_RIGHT_28203				
W1_28F_21996_2	W1_28R_22975_2	W1_64L_22457	W1_63R_22612	W1_36F_28716_2	W1_36R_29724_2	SC2M1-75_LEFT_29344	SC2M1-74_RIGHT_29469				
W1_29F_22864_2	W1_29R_23795_2	W1_66L_23182	W1_65R_23308	W1_37F_29596_2	W1_37R_29873_2						
W1_30F_23704_2	W1_30R_24610_2	W1_69L_24259	W1_67R_24002	0_1b	W1_1R_490						
W1_31F_24514_2	W1_31R_25476_2	W1_71L_24935	W1_70R_25075								
W1_32F_25357_2	W1_32R_26358_2	SC2M1-66_LEFT_25665	SC2M1-65_RIGHT_25790								

Sequencing Reaction – Plate 2
Sequencing Primer Location

Appendix D – Multiplex PCR Primers

Pool 1

PCR	Name	Sequence
1	SC2M1-1_LEFT_31	ACCAACCAACTTTCGATCTCTTGT
	SC2M1-1_RIGHT_574	TGTCTCACCACTACGACCGTAC
5	SC2M1-5_LEFT_1706	TCTGCTTCCACAAGTGCTTTTGT
	SC2M1-5_RIGHT_2266	ACAGGTGACAATTTGTCCACCG
9	SC2M1-9_LEFT_3306	TGGAACTTACACCAGTTGTTCAGAC
	SC2M1-9_RIGHT_3878	CAGCGATCTTTTGTTCAACTTGCT
13	SC2M1-13_LEFT_4885	TCCTACCACATTCCACCTAGATGG
	SC2M1-13_RIGHT_5400	GCACAAAAGTTAGCAGCTTCACC
17	SC2M1-17_LEFT_6408	CTGAAGAAGTAGTGGAAAATCCTACCA
	SC2M1-17_RIGHT_6903	GCCTCTAGACAAAATTTACCGACACT
21	SC2M1-21_LEFT_8004	TTGGTGATAGTGCGGAAGTTGC
	SC2M1-21_RIGHT_8553	CCACCCTTAAGTGCTATCTTTGTTGT
25	SC2M1-25_LEFT_9551	CCAGTTTACTCATTCTTACCTGGTGT
	SC2M1-25_RIGHT_10061	AACCACTCTGCAAAACAGCTGA
29	SC2M1-29_LEFT_11047	AGTCCAGAGTACTCAATGGTCTTTGT
	SC2M1-29_RIGHT_11541	ACAATACCTCTGGCCAAAAACATGA
33	SC2M1-33_LEFT_12557	ATCCAACAGGTTGTAGATGCAGAT
	SC2M1-33_RIGHT_13136	TTGGTTGTCCCCCACTAGCTAG
37	SC2M1-37_LEFT_14103	TTTCATACAAACCACGCCAGGT
	SC2M1-37_RIGHT_14641	GTGCAGCTACTGAAAAGCACGT
41	SC2M1-41_LEFT_15637	AGAAATAGAGATGTTGACACAGACTTTGT
	SC2M1-41_RIGHT_16208	GCCTCATAAAACTCAGGTTCCCA
45	SC2M1-45_LEFT_17317	AATGCATTGCCTGAGACGACAG
	SC2M1-45_RIGHT_17903	CAAGAGTGAGCTGTTTCAGTGGT
49	SC2M1-49_LEFT_18897	TGTTAAGCGTGTTGACTGGACT
	SC2M1-49_RIGHT_19484	GCACCACCTAAATTGCAACGTG
53	SC2M1-53_LEFT_20554	TCTGTAGTTTCTAAGGTTGTCAAAGTGA
	SC2M1-53_RIGHT_21144	AGCTAGCTTTTGTTGTATAAACCCACA
57	SC2M1-57_LEFT_22203	GTGATCTCCCTCAGGGTTTTTCG
	SC2M1-57_RIGHT_22697	ACTTAAAAGTGGAAAATGATGCGGAA
61	SC2M1-61_LEFT_23737	AATTCTACCAGTGTCTATGACCAAGAC
	SC2M1-61_RIGHT_24231	GCACCAAAGGTCCAACCAGAAG
65	SC2M1-65_LEFT_25214	CTAGGTTTTATAGCTGGCTTGATTGC
	SC2M1-65_RIGHT_25790	CATTTCCAGCAAAGCCAAAGCC
69	SC2M1-69_LEFT_26877	CTTCTCAACGTGCCACTCCATG
	SC2M1-69_RIGHT_27432	AGCGAGTGTTATCAGTGCCAAG
73	SC2M1-73_LEFT_28525	TGGCTACTACCGAAGAGCTACC
	SC2M1-73_RIGHT_29045	GCTTCTTAGAAGCCTCAGCAGC

Pool 2

PCR	Name	Sequence
2	SC2M1-2_LEFT_445	TTTGCCTCAACTTGAACAGCCC
	SC2M1-2_RIGHT_965	GTTCACGGCAGCAGTATACACC
6	SC2M1-6_LEFT_2138	AAACCCGTCCTTGATTGGCTTG
	SC2M1-6_RIGHT_2642	TTTCGAGCAACATAAGCCCGTT
10	SC2M1-10_LEFT_3715	AGCTGGTATTTTTGGTGCTGACC
	SC2M1-10_RIGHT_4262	CCTGACCCGGGTAAGTGGTTAT
14	SC2M1-14_LEFT_5258	ACTTCTATTAAATGGGCAGATAACAACTGT
	SC2M1-14_RIGHT_5818	AGCACCGTCTATGCAATACAAAGT
18	SC2M1-18_LEFT_6748	AAACCGTGTTTGTACTAATTATATGCCTT
	SC2M1-18_RIGHT_7255	TGCCAAAAACCACTCTGCAACT
22	SC2M1-22_LEFT_8407	CGTTAAAGATTTCATGTCATTGTCTGAACA
	SC2M1-22_RIGHT_8913	TGCAAAAAGTCACCATTAGTTGTGC
26	SC2M1-26_LEFT_9903	AGTACAAGTATTTTAGTGGAGCAATGGA
	SC2M1-26_RIGHT_10451	TGGGCCTCATAGCACATTGGTA
30	SC2M1-30_LEFT_11400	TGAATGTCTTGACACTCGTTTATAAAGTT
	SC2M1-30_RIGHT_11944	CTGGACACATTGAGCCCACAAT
34	SC2M1-34_LEFT_13006	TGCCACAGTACGTCTACAAGCT
	SC2M1-34_RIGHT_13501	GTGTAAGACGGGCTGCACTTAC
38	SC2M1-38_LEFT_14480	ACTTCAGAGAGCTAGGTGTTGTACA
	SC2M1-38_RIGHT_15027	TGCGAAAAGTGCATCTTGATCCT
42	SC2M1-42_LEFT_16065	GGAGTATGCTGATGTCTTTCATTTGTAC
	SC2M1-42_RIGHT_16648	GCGTTTCTGCTGCAAAAAGCTT
46	SC2M1-46_LEFT_17752	TGGAGAAAAGCTGTCTTTATTTCACCT
	SC2M1-46_RIGHT_18275	GCTTCTTCGCGGGTGATAAACA
50	SC2M1-50_LEFT_19311	TGCATTCCACACACCAGCTTTT
	SC2M1-50_RIGHT_19866	ATTAGCAGCAATGTCCACACCC
54	SC2M1-54_LEFT_20990	TGATTGGTGATTGTGCAACTGTACA
	SC2M1-54_RIGHT_21562	TGTTCGTTTAGTTGTTAACAAGAACATCA
58	SC2M1-58_LEFT_22563	ACTTGTGCCCTTTTGGTGAAGT
	SC2M1-58_RIGHT_23128	TGCTGGTGCATGTAGAAGTTCA
62	SC2M1-62_LEFT_24095	GCTGCTAGAGACCTCATTTGTGC
	SC2M1-62_RIGHT_24623	AAGCTCTGATTTCTGCAGCTCT
66	SC2M1-66_LEFT_25665	CTCACACCTTTTGCTCGTTGCT
	SC2M1-66_RIGHT_26224	GTGCTTACAAAGGCACGCTAGT
70	SC2M1-70_LEFT_27254	TTATGAGGACTTTTAAAGTTTCCATTTGGA
	SC2M1-70_RIGHT_27808	AGCAGAAAGGCTAAAAAGCACAAA
74	SC2M1-74_LEFT_28918	TGATGCTGCTCTTGCTTTGCTG
	SC2M1-74_RIGHT_29469	TCTGCAGCAGGAAGAAGAGTCA
78	SC2M1-52_LEFT2_20349	AGTCAGTTAGGTGGTTTACATCTACTGA
	SC2M1-52_RIGHT2_20798	TTTTGCGACATTCATCATTATGCCT

Pool 3

PCR	Name	Sequence
3	SC2M1-3_LEFT_827	AACAACTTCTGTGGCCCTGATG
	SC2M1-3_RIGHT_1395	TCTGAATTGTGACATGCTGGACA
11	SC2M1-11_LEFT_4126	GGGTGATGTTGTTCAAGAGGGT
	SC2M1-11_RIGHT_4658	ACCGAGCAGCTTCTTCCAAATT
15	SC2M1-15_LEFT_5677	TGTTATGATGTCAGCACCACCTG
	SC2M1-15_RIGHT_6172	AGCCACCACATCACCATTTAAGT
19-2	SC2M1-19b_LEFT_7235	TGCAGAGTGGTTTTTGGCATATATTCT
	SC2M1-19_RIGHT_7694	ACTGTAGTGACAAGTCTCTCGCA
23	SC2M1-23_LEFT_8778	TTAGCCAGCGTGGTGGTAGTTA
	SC2M1-23_RIGHT_9330	TCTACACCACAGAAAACTCCTGGT
27	SC2M1-27_LEFT_10318	GCTTAAGGTTGATACAGCCAATCCT
	SC2M1-27_RIGHT_10837	AACGGCAATTCCAGTTTGAGCA
31	SC2M1-31_LEFT_11810	GGCAAACCTTGTATCAAAGTAGCC
	SC2M1-31_RIGHT_12335	TTGCCCTCTTGTCCTCAGATCT
35	SC2M1-35_LEFT_13366	AAACACAGTCTGTACCGTCTGC
	SC2M1-35_RIGHT_13861	TGTCACAATTACCTTCATCAAAATGCC
39	SC2M1-39_LEFT_14888	ACGATGGTGGCTGTATTAATGCT
	SC2M1-39_RIGHT_15391	GGTGTGACAAGCTACAACACGT
43	SC2M1-43_LEFT_16518	AAATACATGTGTTGGTAGCGATAATGTT
	SC2M1-43_RIGHT_17087	GGTGGTCCCTGGAGTGTAGAAT
47	SC2M1-47_LEFT_18148	GGTTTATGTGTTGACATACCTGGCA
	SC2M1-47_RIGHT_18668	CATAGACAACAGGTGCGCTCAG
51	SC2M1-51_LEFT_19725	TGATGGTGTTGATGTAGAATTGTTTGAA
	SC2M1-51_RIGHT_20255	TCAATTTCCATTTGACTCCTGGGT
55	SC2M1-55_LEFT_21421	AGGGGTACTGCTGTTATGTCTTTAAA
	SC2M1-55_RIGHT_21916	AAGTAGGGACTGGGTCTTCGAA
59	SC2M1-59_LEFT_22986	CCGGTAGCACACCTTGTAATGG
	SC2M1-59_RIGHT_23519	CCCCTATTAAACAGCCTGCACG
63	SC2M1-63_LEFT_24493	AAATGATATCCTTTCACGTCTTGACAAA
	SC2M1-63_RIGHT_25003	TGAGTCTAATTCAGGTTGCAAAGGA
67	SC2M1-67_LEFT_26096	AAAATTGTTGATGAGCCTGAAGAACA
	SC2M1-67_RIGHT_26590	ACTAGGTTCCATTGTTCAAGGAGC
71	SC2M1-71_LEFT_27650	TGTTCATCAGACAAGAGGAAGTTCA
	SC2M1-71_RIGHT_28203	ACGAACAACGCACTACAAGACT
75	SC2M1-75_LEFT_29344	TGACGCATACAAAACATTCCCAC
	SC2M1-75_RIGHT_29848	AAAATCACATGGGGATAGCACTACT

Pool 4

PCR	Name	Sequence
4	SC2M1-4_LEFT_1262	ACGGGCGATTTTGTTAAAGCCA
	SC2M1-4_RIGHT_1840	TCACCAATATTCCAGGCACCTTT
8	SC2M1-8_LEFT_2932	ACTTACACCACTGGGCATTGATT
	SC2M1-8_RIGHT_3461	CTGCAACACCTCCTCCATGTTT
12	SC2M1-12_LEFT_4519	TGGTGCTAGATTTTACTTTTACACCAGT
	SC2M1-12_RIGHT_5017	CACAACTTGCGTGTGGAGGTTA
16	SC2M1-16_LEFT_6030	ACGCAAGCTTCGATAATTTTAAGTTTGT
	SC2M1-16_RIGHT_6544	TGTGTGGCCAACCTCTTCTGTA
20	SC2M1-20_LEFT_7560	GGTCCTTTTATGTCTATGCTAATGGAGG
	SC2M1-20_RIGHT_8128	TGCAAGTTCAGCTTCTGCAGTT
24	SC2M1-24_LEFT_9203	GATTCTGAGTACTGTAGGCACGG
	SC2M1-24_RIGHT_9734	AGAACCAATAGAAATGCTTTGTGGAAA
28	SC2M1-28_LEFT_10697	GGAGACAGGTGGTTTCTCAATCG
	SC2M1-28_RIGHT_11209	AGCTACAGTGGCAAGAGAAGGT
32	SC2M1-32_LEFT_12201	AGTTGAAGAAGTCTTTGAATGTGGCT
	SC2M1-32_RIGHT_12719	TCTGTCGTAGTGCAACAGGACT
36	SC2M1-36_LEFT_13727	GCTGTTGCTAAACATGACTTCTTTAAGT
	SC2M1-36_RIGHT_14232	AGGCTTTGTTAAGTCAGTGTCAACA
40	SC2M1-40_LEFT_15264	TGTAGAAAACCCTCACCTTATGGG
	SC2M1-40_RIGHT_15771	AGCCACTAGACCTTGAGATGCA
44	SC2M1-44_LEFT_16948	CCTACACTAGTGCCACAAGAGC
	SC2M1-44_RIGHT_17458	GTGCAGGTAATTGAGCAGGGTC
48	SC2M1-48_LEFT_18506	GACTTCCTTGGAATGTAGTGCGT
	SC2M1-48_RIGHT_19038	ACCAATGTCGTGAAGAACTGGG
52	SC2M1-52_LEFT_20124	TGGAGAAGCCGTAAAAACACAGT
	SC2M1-52_RIGHT_20698	GATTAGGCATAGCAACACCCGG
56	SC2M1-56_LEFT_21775	TGGGACCAATGGTACTAAGAGGT
	SC2M1-56_RIGHT_22345	ACCAGCTGTCCAACCTGAAGAA
60	SC2M1-60_LEFT_23379	ACCAGGTTGCTGTTCTTTATCAGG
	SC2M1-60_RIGHT_23876	CAGCTATTCCAGTTAAAGCACGGT
64	SC2M1-64_LEFT_24858	GCACACACTGGTTTGTAACACAA
	SC2M1-64_RIGHT_25369	TTTGACTCCTTTGAGCACTGGC
68	SC2M1-68_LEFT_26454	TCCTGATCTTCTGGTCTAAACGAACT
	SC2M1-68_RIGHT_27004	ATGTCACAGCGTCCTAGATGGT
72	SC2M1-72_LEFT_28066	TTGAATTGTGCGTGGATGAGGC
	SC2M1-72_RIGHT_28649	TAGCACCATAGGGAAGTCCAGC
76	SC2M1-1_LEFT2_1	TTAAAGGTTTATACCTTCCCAGG
	SC2M1-1_RIGHT2_495	CGAGCATCCGAACGTTTGATGA

Pool 5

PCR	Name	Sequence
5W	W1_5L_1457	GTAAGGGTGGTCGCACTATTGC
	W1_5R_1969	TTGTTATAGCGGCCTTCTGTAAAAC
81*	SC2M1-19a_LEFT_6957	TGGTTTTTACTATTAAGTGTTTGCCTAGGT
	SC2M1-19a_RIGHT_7393	TCGGGGCCATTTGTACAAGATT
81*	SC2M1-19a_LEFT_6957	TGGTTTTTACTATTAAGTGTTTGCCTAGGT
	SC2M1-19a_RIGHT_7393	TCGGGGCCATTTGTACAAGATT
83	SC2M1-21a_LEFT_7984	AGGCATTAGTGTCTGATGTTGGTG
	SC2M1-21a_RIGHT_8384	TGACTTTTTGCTACCTGCGCAT
28W	W1_28L_9659	TCACACCTTTAGTACCTTTCTGGATAAC
	W1_28R_10207	GGTTAAGCATGTCTTCAGAGGTGC
85	SC2M1-34a_LEFT_12994	GTAGTTTAGCTGCCACAGTACGT
	SC2M1-34a_RIGHT_13399	AACCTTTCCACATACCGCAGAC
42	SC2M1-42_LEFT_16065	GGAGTATGCTGATGTCTTTCATTTGTAC
	SC2M1-42_RIGHT_16648	GCGTTTCTGCTGCAAAAAGCTT
89	SC2M1-49a_LEFT_18711	CCTGTTGGCATCATTCTATTGGATTT
	SC2M1-49a_RIGHT_19112	GTCACTACAAGGCTGTGCATCA
91	SC2M1-50a_LEFT_19181	TGCCTATTTTGGAATTGCAATGTCG
	SC2M1-50a_RIGHT_19569	AAACCCACAAGCTAAAGCCAGC
93	SC2M1-51a_LEFT_19661	TTTGATGGACAACAGGGTGAAGT
	SC2M1-51a_RIGHT_20098	GCTTGTTTGGGACCTACAGATGG
60W	W1_60L_21029	GGATCTCATTATTAGTGATATGTACGACCC
	W1_60R_21562	TTGTTCGTTTAGTTGTTAACAAGAACATC
64W	W1_64L_22457	CAAAGTGTACGTTGAAATCCTTCACTG
	W1_64R_22993	TGCTACCGGCCTGATAGATTTC
98	SC2M1-67a_LEFT_25910	GGCACAACAAGTCCTATTTCTGAAC
	SC2M1-67a_RIGHT_26276	CGTACCTGTCTCTTCCGAAACG
100	SC2M1-69a_LEFT_26846	TGTGGTCATTCAATCCAGAAACTAACA
	SC2M1-69a_RIGHT_27226	ACCTGAAAGTCAACGAGATGAAACA
102	SC2M1-70a_LEFT_27252	TTATGAGGACTTTTAAAGTTTCCATTTGGA
	SC2M1-70a_RIGHT_27644	AGGTGAAACTGATCTGGCACGT
71	SC2M1-71_LEFT_27650	TGTTCATCAGACAAGAGGAAGTTCA
	SC2M1-71_RIGHT_28203	ACGAACAACGCACTACAAGACT
95	0_1b	TTAAAGGTTTATACCTTCCCAGGTA
	W1_1R_490	CATCCGAACGTTTGATGAACAC
36	SC2M1-36_LEFT_13727	GCTGTTGCTAAACATGACTTCTTTAAGT
	SC2M1-36_RIGHT_14232	AGGCTTTGTTAAGTCAGTGTCAACA

Pool 6

PCR	Name	Sequence
6W	W1_6L_1819	AGGTGCCTGGAATATTGGTGAAC
	W1_6R_2345	ATGATAGAGTCAGCACACAAAGC
7ab	SC2M1-7a_LEFT_2491	AGGGAGAAACACTTCCCACAGA
	SC2M1-7b_RIGHT_3165	AGCAGAAGTGGCACCAAATTCC
84	SC2M1-21b_LEFT_8240	TCAATCTGACATAGAAGTTACTGGCG
	SC2M1-21b_RIGHT_8618	GCAGCAACAAAAAGGAACACAAGT
26W	W1_26L_8999	CTTGTGTTTTGGCTGCTGAATG
	W1_26R_9459	CATAAAATAGTAGGCAAGGCATGTTACTAC
40W	W1_40L_13986	CGCCAAGCTTTGTTAAAAACAGTAC
	W1_40R_14503	TGTACAACACCTAGCTCTCTGAAGTG
86	SC2M1-34b_LEFT_13245	CTGTACTGCCGTTGCCACATAG
	SC2M1-34b_RIGHT_13620	CGTCCTTTTCTTGGAAGCGACA
90	SC2M1-49b_LEFT_18955	TGCGGCTTGTAGAAAGGTTCAA
	SC2M1-49b_RIGHT_19331	AAAAGCTGGTGTGTGGAATGCA
92	SC2M1-50b_LEFT_19395	AGTCTCATGGAAAACAAGTAGTGTCA
	SC2M1-50b_RIGHT_19820	TGGTACTGGTTTAATGTTGCGCT
94	SC2M1-51b_LEFT_19957	AACGATTTGTGCACCACTCACT
	SC2M1-51b_RIGHT_20373	TCAGTAGATGTAAACCACCTAACTGACT
99	SC2M1-67b_LEFT_26128	TCACACAATCGACGGTTCATCC
	SC2M1-67b_RIGHT_26541	GTACCGTTGGAATCTGCCATGG
101	SC2M1-69b_LEFT_27080	TAGCAGGTGACTCAGGTTTTGC
	SC2M1-69b_RIGHT_27443	AAGCTCACAAGTAGCGAGTGTT
9	SC2M1-9_LEFT_3306	TGGAACTTACACCAGTTGTTCAGAC
	SC2M1-9_RIGHT_3878	CAGCGATCTTTTGTTCAACTTGCT
75	SC2M1-75_LEFT_29344	TGACGCATACAAAACATTCCCAC
	SC2M1-75_RIGHT_29848	AAAATCACATGGGGATAGCACTACT
70b	SC2M1-70b_LEFT_27497	TCTTCTGGAACATACGAGGGCA
	W1_34R_28006_1	AGGACACGGGTCATCAACTAC
95	0_1b	TTAAAGGTTTATACCTTCCCAGGTA
	W1_1R_490	CATCCGAACGTTTGATGAACAC
19-3	SC2M1-19b_LEFT_7235	TGCAGAGTGGTTTTTGGCATATATTCT
	W1_21R_7771	GATGGATGGAACCATTCTTCACTG
62-2	SC2M1-62a1_LEFT_23993	ACCAAGCAAGAGGTCATTTATTGAAGA
	W1_30R_24625_1	CAGAAGCTCTGATTTCTGCAGC

*This amplicon is intentionally doubled due to its reduced efficiency—add 2 parts of this primer set to the pool.

Appendix E – AMPure XP bead clean-up

Bead-based clean-ups are done at several steps throughout the protocols presented. This covers the basic clean-up steps, make sure to check the specific protocol for the ratio of beads to use.

Depending on the number of samples, the AMPure XP bead clean-up takes about 30-40 minutes.

Required reagents for bead-based clean-up

Company	Product	Catalog number
Beckman Coulter	Agencourt AMPure XP beads	A63882
	10mM Tris-HCl pH 8.0	

1. Allow AMPure XP beads to warm to room temperature for at least 30 minutes before using.
2. Vortex AMPure XP beads to re-suspend.
3. Add appropriate ratio of re-suspended AMPure XP beads to the ligation reaction. Mix well by pipetting up and down at least 10 times.
4. Incubate for 5 minutes at room temperature.
5. Place the tube/plate on an appropriate magnetic stand to separate the beads from the supernatant. If necessary, quickly spin the sample to collect the liquid from the sides of the tube or plate wells before placing on the magnetic stand.
6. After the solution is clear (about 5 minutes), carefully remove and discard the supernatant. Be careful not to disturb the beads that contain DNA targets (do not discard beads).
7. Add 200 uL of freshly prepared 80% Ethanol to the tube/plate while in the magnetic stand.
8. Incubate at room temperature for 30 seconds, then carefully remove and discard the supernatant.
9. Add another 200 uL of freshly prepared 80% Ethanol to the tube/plate while in the magnetic stand.
10. Incubate at room temperature for 30 seconds, then carefully remove and discard the supernatant.
11. Air dry the beads for 2 minutes while the tube/plate is on the magnetic stand and with the lid(s) open.

    Caution: Do not over dry the beads. This may result in lower recovery of DNA target.
12. Remove the tube/plate from the magnet. Elute the DNA target from the beads by adding appropriate volume of 10mM Tris-HCl or water.
13. Mix well by pipetting up and down or on a vortex mixer. Incubate for 2 minutes at room temperature. If necessary, quickly spin the sample to collect liquid from the sides of the tube or plate wells before placing on the magnetic stand.
14. Place the tube/plate on the magnetic stand.
15. After the solution is clear (about 5 minutes), transfer to a new tube.

Appendix F – Quantitation using Qubit

Quantitation is done at several various steps throughout the protocols included and this protocol can be used anytime quantitation is indicated.

Required reagents

Company	Product	Catalog number
Thermo Fisher	dsDNA HS assay kit	Q32854
Thermo Fisher	dsDNA BR assay kit	Q32850
Thermo Fisher	Qubit assay tubes	Q32856

Note: depending on the sample, either the high sensitivity (HS) or broad range (BR) kit may be used, the protocols are the same the only difference is the reagents.

Quantitation takes about 10-20 minutes depending on the number of samples.

Procedure

1. Set up the required number 0.5 mL Qubit assay tubes for standards and samples. Note: the standards require two tubes.
2. Label tube lids. Do not label the side of the tube as this could interfere with the sample read.
3. Prepare the Qubit working solution by diluting the Qubit dsDNA HS reagent 1:200 in Qubit dsDNA HS buffer. Use a clean plastic tube each time you prepare Qubit working solution. Do not mix the working solution in a glass container.

    The final volume in each tube must be 200 uL. Each standard tube requires 190 uL of Qubit working solution and each sample tube requires anywhere from 180-199 uL. Prepare sufficient Qubit working solution to accommodate all standards and samples.

4. Add 190 uL of Qubit working solution to each of the tubes used for standards.
5. Add 10 uL of each qubit standard to the appropriate tube, mix by vertexing 2-3 seconds.
6. Add Qubit working solution to individual assay tube, mix by vertexing 2-3 seconds.

    Your sample can be anywhere from 1-20 uL. Add a corresponding volume of Qubit working solution to each assay tube: anywhere from 180-199 uL.

7. Add each sample to the assay tubes containing the correct volume of Qubit working solution, then mix by vertexing 2-3 seconds. The final volume in each tube should be 200 uL.
8. Allow all tubes to incubate at room temperature for 2 minutes.
9. Sample concentration can now be measured on the Qubit Fluorometer.

Appendix G – CENTRI-SEP 96 Protocol

PRINCETON
SEPARATIONS

CENTRI-SEP 96
Protocol

CENTRI•SEP 96 plates must be allowed to equilibrate to room temperature before use. We recommend that the plates be removed from the refrigerator at the same time the sequencing reactions are initiated. This will allow sufficient time for the plates to warm.

1. Remove the adhesive foil from the bottom and then from the top of the CENTRI-SEP 96 plate.
2. Stack the CENTRI-SEP 96 plate on top of a 96-well wash plate and centrifuge at 1500 x g for 2 minutes. Use an external timer and start timing when the rotor has reached the set speed. Discard the liquid in the wash plate. The gel matrix in the wells should appear opaque at this point.
3. Transfer the samples (20 µL or less) to the individual wells in the CENTRI-SEP 96 plate, taking care to place the samples in the centers of the gel beds.
4. Stack the CENTRI-SEP 96 plate on top of a 96-well collection plate and centrifuge at 1500 x g for 2 minutes.
5. Remove the 96-well collection plate containing the cleaned samples and dry in a speed-vac equipped with the appropriate rotor. Alternatively the plate can be sealed for storage.
